# An efficient preparation and biocatalytic synthesis of novel *C*-glycosylflavonols kaempferol 8-*C*-glucoside and quercetin 8-*C*-glucoside through using resting cells and macroporous resins

**DOI:** 10.1186/s13068-022-02228-5

**Published:** 2022-11-24

**Authors:** Yangbao Wu, Huan Wang, Yang Liu, Linguo Zhao, Jianjun Pei

**Affiliations:** 1 Jiangsu Co-Innovation Center of Efficient Processing and Utilization of Forest Resources, College of Chemical Engineering, Nanjing Forestry University, Nanjing, 210037 China; 2 Jiangsu Key Lab of Biomass-Based Green Fuels and Chemicals, Nanjing, 210037 China

**Keywords:** Kaempferol 8-*C*-glucoside, Quercetin 8-*C*-glucoside, UDP-glucose biosynthesis pathway, Resting cell bioconversion, Adsorption/desorption, Metabolic engineering

## Abstract

**Background:**

*C-*glycosylated flavonoids are a main type of structural modification and can endow flavonoids with greater stability, bioactivity, and bioavailability. Although some *C*-glycosylated flavonoids have been biosynthesized in vivo or vitro, only a few *C*-glycosylflavonols have been prepared by these methods.

**Results:**

In this study, several uridine 5’-diphosphate (UDP)-glucose biosynthesis pathways and *Escherichia coli* hosts were screened to reconstruct recombinant strains for producing the novel *C*-glycosylflavonols kaempferol 8-*C*-glucoside and quercetin 8-*C*-glucoside. To increase *C*-glycosylflavonol production, the timing of flavonol addition was adjusted, and glycerol was added to avoid degradation of *C*-glycosylflavonols. By using resting cell bioconversion, the highest kaempferol 8-*C*-glucoside and quercetin 8-C-glucoside production reached 16.6 g/L and 12.5 g/L, respectively. Then, ultrasound-assisted adsorption/desorption was used to prepare *C*-glycosylflavonols by using macroporous resins. Through screening macroporous resins and optimizing the adsorption/desorption conditions, the highest adsorption capacity and desorption capacity for kaempferol 8-*C*-glucoside on HPD100 reached 28.57 mg/g and 24.15 mg/g, respectively. Finally, kaempferol 8-*C*-glucoside (15.4 g) with a yield of 93% and quercetin 8-*C*-glucoside (11.3 g) with a yield of 91% were obtained from 1 L of fermentation broth.

**Conclusions:**

Kaempferol 8-*C*-glucoside and quercetin 8-*C*-glucoside are novel *C*-glycosylflavonols, which have not been extracted from plants. This study provides an efficient method for the preparation and biocatalytic synthesis of kaempferol 8-*C*-glucoside and quercetin 8-*C*-glucoside by metabolic engineering of *Escherichia coli*.

**Graphical Abstract:**

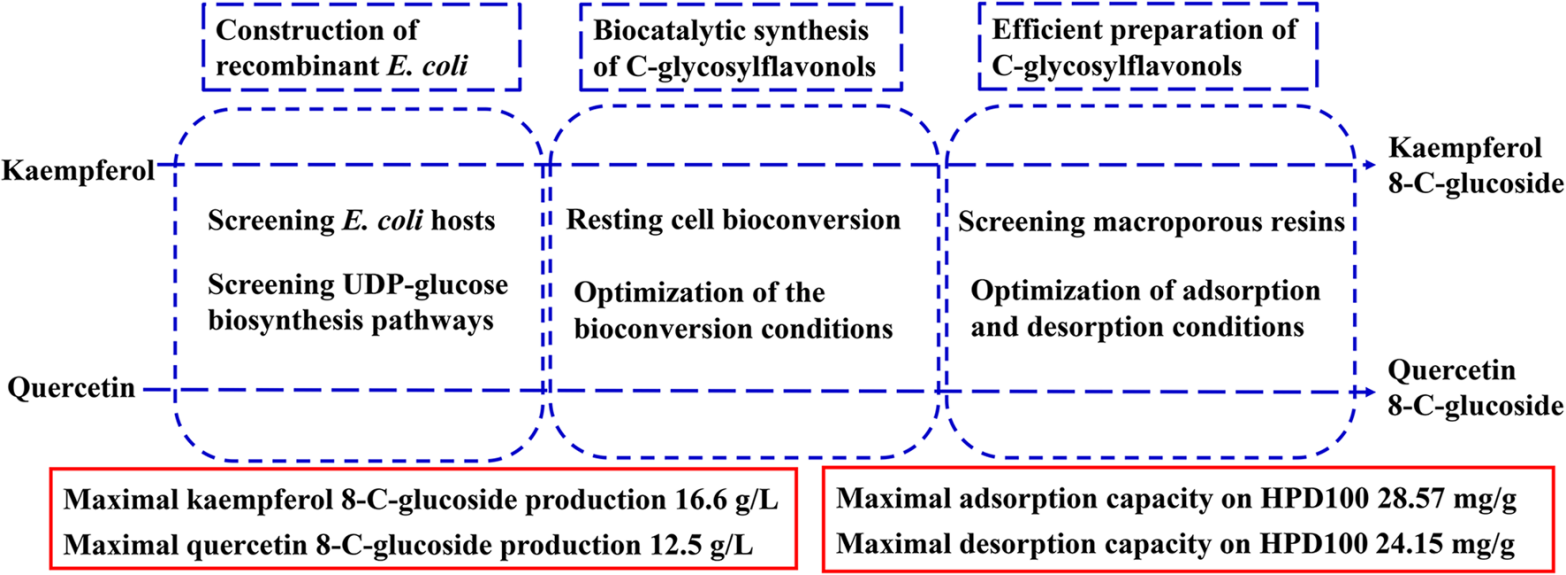

**Supplementary Information:**

The online version contains supplementary material available at 10.1186/s13068-022-02228-5.

## Introduction

Flavonoids, including flavanones, flavones, flavonols, and anthocyanidins, are an important class of secondary metabolites found in plants and have a variety of physiological activities [1–6]. More than 10,000 flavonoids have been characterized from various plants according to their chemical structures [7, 8]. In nature, glycosylation of flavonoids is an important type of structural modification, and modifications can not only improve their solubility and stability but also impart specific activity, selectivity and pharmacological properties [9–12]. *C*-glycosylated flavonoids are more stable to hydrolysis by acid and glycosidase than *O*-glycosylated flavonoids, which have been found in various food crops and health products, such as wheat, maize, rice, mung bean, blueberry, acai pulp, rooibos tea, lemon juice, and bamboo leaf extract [13–19]. Several *C*-glycosylated flavonoids, such as *C*-glycosylflavones (vitexin, orientin, isovitexin, and isoorientin), *C*-glycosyldihydrochalcone (nothofagin), and *C*-glycosylchromone (aloesin), have been extracted from plants and reported to display antioxidant, anti-insect, antimicrobial, antidiabetic, antitumorigenic, antinociceptive hepatoprotective, and anti-inflammatory activities [19, 20].

Flavonols and their *O*-glycosyl derivatives are significant derivatives of flavonoids. Some *O*-glycosylflavonols have been extracted from traditional medicines in East Asia. For example, quercetin 3-*O*-galactoside (hyperoside) and kaempferol 3-*O*-glucoside (astragalin) are used as marker compounds to assess the quality of *Hyperin perforatum L.* and *Radix astragali* extracts, respectively [21, 22]. Some recombinant strains and one-pot enzymatic cascades have been developed to biosynthesize *O*-glycosylflavonols in vivo and in vitro [11, 23, 24]. Although many kinds of *O*-glycosylflavonols have been biosynthesized, only a few *C*-glycosylflavonols have been prepared by these methods [25].

In plants, *C*-glycosylated flavonoids are synthesized by *C*-glycosyltransferases, which transfer an activated sugar (UDP-sugar) to an acceptor molecule (flavonoids). Some *C*-glycosyltransferase genes have been cloned, expressed, and characterized from different plants [26–33]. Most *C*-glycosyltransferases can only catalyze the *C*-glycosylation with the open-chain form of 2-hydroxyflavanone as the substrate [27, 28, 33]. The recombinant strains were successfully reconstructed by introducing the *Oryza sativa C*-glycosyltransferase (OsCGT) gene to biosynthesize *C*-glycosylated flavones with a production yield of 7.2 mg/L [34]. Bungaruang et al. developed an efficient enzymatic cascade for nothofagin production by using the coupled catalysis of OsCGT and *Glycine max* sucrose synthase [35]. Compared to the above *C*-glycosyltransferases, *Gentiana triflora C*-glycosyltransferase *C*-glycosylates flavones with the flavone as the substrate [29]. Based on the enzyme, some recombinant strains and enzymatic cascades were developed to produce *C*-glycosylated flavones (isoorientin and isovitexin) [36]. In recent years, a *C*-glycosyltransferase (TcCGT) gene, which can *C*-glycosylate flavonols, was cloned from *Trollius chinensis* [32, 37]. The identification of *TcCGT* made it possible to reconstruct the recombinant strain to produce *C*-glycosylflavonols.

Metabolic engineering is an effective method for the production of glycosylated flavonoids. The recombinant strains were reconstructed by introducing glycosyltransferase genes and UDP-sugar synthesis pathways to improve production. De Bruyn et al. developed an effective glycosylation platform in *E. coli* W strain by introducing the *Bifidobacterium adolescentis* sucrose phosphorylase (Basp) gene and the *B. bifidum* uridylyltransferase (UgpA) gene [23]. Subsequently, two recombinant *E. coli* W strains were engineered by reconstructing the UDP-galactose synthesis pathway and UDP-rhamnose pathway to produce hyperoside (0.94 g/L) and quercitrin (1.12 g/L) [11]. We reconstructed recombinant strains to produce isoorientin by introducing the *Gentiana triflora C*-glycosyltransferase gene and the cellobiose phosphorolysis pathway, and isoorientin production reached 1371 mg/L [36]. Although some metabolic engineering approaches have been developed to reconstruct recombinant strains for biosynthesizing stereospecific glycosylated flavonoids, the yield of glycosylated flavonoids is unsuitable for large-scale applications.

Flavonoids, as antimicrobial agents, can inhibit the growth of *E. coli* by inhibiting the activity of DNA topoisomerase [38, 39]. The inhibitory effects of flavonoids improved substantially with increasing production. Therefore, the separation of growth and transformation for *E. coli* is important to improve the production of flavonoids. Resting cell bioconversion has been developed to biosynthesize secondary metabolites, as it can overcome these problems [40, 41]. Moreover, some flavonoid-degrading enzyme genes exist in the genome of *E. coli* and can be induced under certain growth conditions. YhhW is a nuclear protein that has the activity of unique quercetin 2,4-dioxygenase and can degrade various flavonoids [42]. The BglA and BglB genes encode phospho-β-glucosidases capable of hydrolyzing glycosylated flavonoids [43]. Thus, avoiding degradation and inhibition effects are key factors for improving the yield of glycosylated flavonoids.

In this study, several UDP-glucose biosynthesis pathways and *E. coli* hosts were screened to reconstruct recombinant strains for producing novel *C*-glycosylflavonols (kaempferol 8-*C*-glucoside and quercetin 8-*C*-glucoside) from kaempferol and quercetin, respectively. To improve the production of *C*-glycosylflavonols, the effects of adding carbon sources to prevent the degradation of *C*-glycosylflavonols and the fermentation conditions of resting cells were determined. Finally, the extraction process of *C*-glycosylflavonols with macroporous resins was also determined.

## Materials and methods

### Strains, plasmids, media, and chemicals

All plasmids and strains used in this research are listed in Table 1. The strains were grown at 37 °C in Luria–Bertani (LB) medium supplemented with antibiotics when needed. Kaempferol and quercetin were purchased from Shanxi Huike Botanical Development Co., Ltd. (Xian, China). Macroporous resins (NKA-9, DM301, AB-8, D101, HPD100, and HP-20) were purchased from Shanghai Yuanye Bio-Technology Co., Ltd. (Shanghai, China), and the physical properties of the macroporous resins can be found in Additional file 1: Table S1. TB medium (12 g/L tryptone, 24 g/L yeast extract, 2.32 g/L KH_2_PO_4_, 12.54 g/L K_2_HPO_4_) containing 10 g/L glycerol (TB-Gly), 10 g/L glucose (TB-Glc), 10 g/L dextrin (TB-Dex), 10 g/L maltodextrin (TB-Mal), or 10 g/L inulin (TB-Inu) was prepared. M9-Gly medium (0.5 g/L NH_4_Cl, 3 g/L Na_2_HPO_4_, 1.5 g/L KH_2_PO_4_, 1 mM MgSO_4_, 1 mM CaCl_2_, and 10 g/L glycerol) was also prepared. The cellobiose phosphorylase gene (*cep*) was obtained from pACYCDuet-Cep-UgpA by using *Nco*I and *EcoR*I digestion and subcloned into pACYCDuet-cscB-Basp-UgpA at the *Nco*I and *EcoR*I sites to create pACYCDuet-cscB-Cep-UgpA.

**Table 1:** Plasmids and strains used in this study

Plasmids/strains	Descriptions	References
Plasmids		
pCDFDuet-TcCGT	pCDFDuet-1 carrying *TcCGT* from *Trollius chinensis,* T7 promoter	[32]
pACYCDuet-Cep-UgpA	pACYCDuet-1 carrying *cep* from *Saccharophagus degradans* and UTP-glucose-1-phosphate uridylyltransferase gene (*ugpA*) from *Bifidobacterium bifidum,* T7 promoter	[36]
pACYCDuet-cscB-Cep-UgpA	pACYCDuet carrying sucrose permease gene (*cscB*) from *E. coli* W, *cep* from *Saccharophagus degradans* and UTP-glucose-1-phosphate uridylyltransferase gene (*ugpA*) from *Bifidobacterium bifidum,* T7 promoter	This study
pACYCDuet-cscB-Basp-UgpA	pACYCDuet carrying sucrose permease gene (*cscB*) from *E. coli* W, sucrose phosphorylase gene (*Basp*) from *B. adolescentis*, and *ugpA* from *B. bifidum,* T7 promoter	[24]
Strains		
BL21 (DE3)	*E. coli B F*^*−*^* dcm ompT hsdS* (r_B_- m_B_-) *gal [malB* +*]*_*K-12*_*(λ*^*S*^*)*	Weidi Biotechnology
BL21 Star (DE3)	*E. coli* F^−^ *omp*T *hsd*S_B_ (r_B_- m_B_-) *gal dcm rne*131 (DE3)	Weidi Biotechnology
JM109 (DE3)	*E. coli end*A1 *rec*A1 *gyr*A96 thi-1 *hsd*R17 (r_k_-,m_k_ +) *rel*A1 *sup*E44 D *(lac-proAB) [*F´ *traD36 pro*AB *laqI *^*q*^*ZΔ*M15*]*(DE3)	Weidi Biotechnology
ER2566	*E. coli F*^*−*^* omp*T *hsd*S_B_ (r_B_- m_B_-) *gal dcm lac*Y1 (DE3)	Weidi Biotechnology
BL-TcCGT	BL21 (DE3) harboring pCDFDuet-TcCGT	This study
BLStar-TcCGT	BL21 Star (DE3) harboring pCDFDuet-TcCGT	This study
JM109-TcCGT	JM109 (DE3) harboring pCDFDuet-TcCGT	This study
ER2566-TcCGT	ER2566 harboring pCDFDuet-TcCGT	This study
BL-TcCGT-I	BL21 (DE3) harboring pCDFDuet-TcCGT and pACYCDuet-cscB-Cep-UgpA	This study
BLStar-TcCGT-I	BL21 Star (DE3) harboring pCDFDuet-TcCGT and pACYCDuet-cscB-Cep-UgpA	This study
JM109-TcCGT-I	JM109 (DE3) harboring pCDFDuet-TcCGT and pACYCDuet-cscB-Cep-UgpA	This study
ER2566-TcCGT-I	ER2566 harboring pCDFDuet-TcCGT and pACYCDuet-cscB-Cep-UgpA	This study
BL-TcCGT-II	BL21 (DE3) harboring pCDFDuet-TcCGT and pACYCDuet-cscB-Basp-UgpA	This study
BLStar-TcCGT-II	BL21 Star (DE3) harboring pCDFDuet-TcCGT and pACYCDuet-cscB-Basp-UgpA	This study
JM109-TcCGT-II	JM109 (DE3) harboring pCDFDuet-TcCGT and pACYCDuet-cscB-Basp-UgpA	This study
ER2566-TcCGT-II	ER2566 harboring pCDFDuet-TcCGT and pACYCDuet-cscB-Basp-UgpA	This study

### C-glycosylflavonol production by the recombinant strains

The recombinant strains were inoculated in 5 mL of TB-Gly medium containing the appropriate antibiotics and grown at 37 °C until the OD_600_ reached 0.8. A total of 2 g/L kaempferol, 5 g/L cellobiose or sucrose, and 0.1 mM IPTG were added to the recombinant strains, and the fermentation broth was incubated at 30 °C with shaking at 180 rpm for 24 h. 2 g/L kaempferol is insoluble and dispersed in medium. Five volumes of methanol were added to the fermentation broth, and the supernatant was harvested by centrifugation at 12,000×*g* for 5 min and analyzed using high-performance liquid chromatography (HPLC).

The effects of the media (LB and different TB media), induction temperature (18, 25, 30 and 37 °C), IPTG concentration (0, 0.01, 0.02, 0.04, 0.08, 0.1, and 0.4 mM), cellobiose concentration (2.5, 5, 10, 15, and 20 g/L), glycerol concentration (5, 10, 15, 20, and 25 g/L), DMSO concentration (1, 2, 4, 6, 8, and 10%), kaempferol concentration (0.8, 1.6, 2.4, 3, and 3.6 g/L), and timing of kaempferol addition (0, 2, 4, 6, 8, 10, and 12 h) on kaempferol 8-*C*-glucoside production were determined. Five volumes of methanol were added to the fermentation broth, and the samples were measured using HPLC.

### Effects of glycerol on avoiding degradation of C-glycosylflavonols

The recombinant strains (BL-TcCGT-I) were added to 200 mL of fresh medium in 1-L shake flasks containing 50 µg/mL streptomycin and 35 µg/mL chloramphenicol and grown at 37 °C until the OD_600_ reached 0.8. The broth was incubated at 20 °C for 10 h after adding 0.02 mM IPTG. Then, 3 g/L quercetin and 10 g/L cellobiose were added to the broth for 54 h of fermentation. In the fermentation process, 10 g/L glycerol was added to the broth at 30 h. Five volumes of methanol were added to the fermentation broth, and the samples were measured using HPLC.

### Kaempferol 8-C-glucoside production by resting cells

The recombinant strains were inoculated into 5 mL of fresh LB medium containing appropriate antibiotics and were grown at 37 °C for 24 h. The seed culture was then inoculated in 250 mL of fresh LB medium containing appropriate antibiotics and was grown at 37 °C until the OD_600_ reached 0.8. After that, IPTG (0.02 mM) was added to the recombinant strains, and the broth was incubated at 20 °C for 12 h. The recombinant strains were harvested by centrifugation at 5000×*g* for 10 min and resuspended in LB, TB-Gly, or M9-Gly medium. Different concentrations of recombinant strains were incubated by using the broth (5 mL) in tubes at 30 °C for 24 h. Then, fifty volumes of methanol were added to the fermentation broths, and the samples were measured using HPLC.

The recombinant strains were inoculated using a method similar to that described above. The recombinant strains (OD_600_ = 40) were incubated by using M9-Gly medium (250 mL) in 1-L triangular flask. A total of 4 g/L kaempferol or quercetin, 10 g/L cellobiose, 5% DMSO, 0.02 mM IPTG, 50 µg/mL streptomycin, and 35 µg/mL chloramphenicol were added to the broth incubated at 30 °C for 120 h. In the fermentation process, 4 g/L kaempferol or quercetin and 10 g/L cellobiose were added to the broth at 24 h and 72 h, and 10 g/L glycerol was added to the broth at 24 h, 48 h, 72 h, and 96 h. Then, fifty volumes of methanol were added to the fermentation broths, and the samples were measured using HPLC.

### Screening of macroporous resins

For the purification of *C*-glycosylflavonols with macroporous resins, the adsorption/desorption capacity of different macroporous resins was determined. Kaempferol 8-*C*-glucoside dissolved in 5% DMSO. NKA-9, DM301, AB-8, D101, HPD100 or HP-20 (1 g dry weight) was mixed with 30 mL of kaempferol 8-*C*-glucoside (1019.7 mg/L) in a 100-mL conical flask under shaking at 80 rpm and 25 °C. After 3 h of adsorption, the resins were washed with distilled water. Then, the adsorbed resins and 30 mL of 50% ethanol were added to each conical flask and placed on a shaker (80 rpm) for 2 h at 25 °C. The adsorption/desorption capacity, ratio, and recovery for each resin were calculated using the following equation:

$${\text{Adsorption capacity }}\left( {{{{\text{mg}}} \mathord{\left/ {\vphantom {{{\text{mg}}} {\text{g}}}} \right. \kern-\nulldelimiterspace} {\text{g}}}{\text{dry resins}}} \right)\, = \,\left( {C_{0} - C_{{\text{e}}} } \right) \times {{V_{0} } \mathord{\left/ {\vphantom {{V_{0} } W}} \right. \kern-\nulldelimiterspace} W},$$

$${\text{Adsorption rate }}\left( \% \right)\, = {{\left( {C_{0} - C_{{\text{e}}} } \right)} \mathord{\left/ {\vphantom {{\left( {C_{0} - C_{{\text{e}}} } \right)} {C_{0} }}} \right. \kern-\nulldelimiterspace} {C_{0} }}\, \times \,{1}00\% ,$$

$${\text{Desorption capacity }}\left( {{\text{mg}}/{\text{g dry resins}}} \right)\, = \,C_{{\text{d}}} \, \times \,V_{{\text{d}}} /W,$$

$${\text{Desorption rate }}\left( \% \right)\, = \,{{C_{{\text{d}}} \, \times \,V_{{\text{d}}} } \mathord{\left/ {\vphantom {{C_{{\text{d}}} \, \times \,V_{{\text{d}}} } {\left( {\left( {C_{0} - C_{{\text{e}}} } \right)\, \times \,V_{0} } \right)}}} \right. \kern-\nulldelimiterspace} {\left( {\left( {C_{0} - C_{{\text{e}}} } \right)\, \times \,V_{0} } \right)}} \times \,{1}00\% ,$$

$${\text{Recovery rate }}\left( \% \right)\, = \,{{C_{{\text{d}}} } \mathord{\left/ {\vphantom {{C_{{\text{d}}} } {C_{0} }}} \right. \kern-\nulldelimiterspace} {C_{0} }} \times \,{1}00\% ,$$

where *C*_0_, *C*_e_, and *C*_d_ represent the initial, equilibrium and desorption solution concentrations (mg/L) of kaempferol 8-*C*-glucoside, respectively; *V*_0_ and *V*_d_ represent the initial kaempferol 8-*C*-glucoside volume and the desorption solution volume (mL), respectively; and W represents the dry weight (g) of the macroporous resin.

### Ultrasound-assisted adsorption and desorption process

Ultrasound-assisted adsorption/desorption processes were performed in a CNC ultrasonic cleaner (KQ-400DE, Kunshan Ultrasonic Instrument Co., Ltd. Kunshan, China). The effects of ultrasound power (240 or 280 W) on the adsorption behaviors of macroporous resins were determined at 25 °C. In the desorption experiment, the effects of ultrasound power (240 and 280 W) and ethanol concentration (40–100%) on the desorption capacity of kaempferol 8-*C*-glucoside were tested. The control group (*P* = 0 W) denoted the sample with shaking treatment (80 rpm) at 25 °C. The diagrams for the adsorption and desorption of *C*-glycosylflavonols via the macroporous resins are shown in Additional file 1: Figure S1.

### Adsorption kinetics

Three adsorption kinetic models, the pseudofirst-order, pseudosecond-order, and particle diffusion models, were used to study the adsorption process:

$${\text{Pseudofirst - order kinetic model}}:ln\left( {Q_{e} \, - \,Q_{t} } \right)\, = \,lnQ_{e} \, - \,K_{0} t,$$

$${\text{Pseudosecond - order kinetic model}}:t/Q_{t} \, = \,{1 \mathord{\left/ {\vphantom {1 {\left( {K_{{1}} Q_{e}^{2} } \right)}}} \right. \kern-\nulldelimiterspace} {\left( {K_{{1}} Q_{e}^{2} } \right)}}\, + \,t/Q_{e} ,$$

$${\text{Particle diffusion model}}:Q_{t} \, = \,K_{{2}} t^{{{1}/{2}}} \, + \,M,$$

where *K*_0_, *K*_1_, and *K*_2_ represent the rate constants for the pseudofirst-order model, pseudosecond-order model, and particle diffusion model, respectively; *Q*_e_ represents the adsorption capacity at equilibrium; *Q*_t_ represents the adsorption capacity at time t; and M indicates the constant in the particle diffusion model.

### Statistical analysis

Data are expressed as mean ± SD. Student’s *T*-test and one-way ANOVA test were used for statistical analyses of the data. All statistical analyses were conducted using SPSS 10.0 statistical software (SPSS, Chicago, IL). Cases in which *P* values of < 0.01 were considered statistically significant.

### HPLC analysis

HPLC analysis of kaempferol, quercetin, kaempferol 8-*C*-glucoside and quercetin 8-*C*-glucoside was performed using an HPLC 1260 system (Agilent, USA) equipped with a C18 column (250 mm × 4.6 mm; i.d., 5 μm) with methanol (A) and distilled water (B) at an A/B ratio of 55:45 for 21 min. The flow rate was set at 0.8 mL/min, and the analytes were detected by monitoring the absorbance at 368 nm.

### Product purification and structural identification

Ten volumes of 5% DMSO were added to the fed-batch reaction broth to dissolve the product. The fed-batch reaction broth was harvested by centrifugation at 8,000 × g for 15 min. The supernatant was mixed with HPD100 macroporous resin (600 g) under shaking treatment (80 rpm and 25 °C) for 3 h and eluted with 80% ethanol under ultrasound power 240 W for 1 h. The eluate was collected and evaporated to dryness, and the product was analyzed by LC/MS and NMR.

LC/MS analysis of kaempferol, quercetin, kaempferol 8-*C*-glucoside and quercetin 8-*C*-glucoside was performed using an LTQ Orbitrap XL LC/MS system in negative mode and an ion trap analyzer (Thermo Fisher Scientific, Waltham, MA, USA). The ion spray was generated at 25 Arb N2/min, 3.5 kV, and 300 °C. ^1^H-NMR and ^13^C-NMR spectra of the purified product were recorded on a Bruker 400 MHz Avance NEO spectrometer (Bruker BioSpin, Switzerland), and DMSO-*d*_*6*_ was used as the solvent.

Kaempferol 8-*C*-glucoside ^1^H NMR (DMSO-*d*_*6*_, 400 MHz): *δ* 12.70 (s, 1H), 10.77 (s, 1H), 10.10 (s, 1H), 9.38 (s, 1H), 8.18 (d, *J* = 8.4 Hz, 2H), 6.90 (d, *J* = 8.3 Hz, 2H), 6.28 (s, 1H), 5.04–4.92 (m, 2H), 4.72–4.55 (m, 3H), 3.90 –3.43 (m, 4H), 3.25 (t, *J* = 8.1 Hz, 2H).

Kaempferol 8-*C*-glucoside ^13^C NMR (DMSO-*d*_*6*_, 101 MHz): δ 176.59, 162.70, 160.14, 159.68, 155.33, 147.51, 135.91, 130.49, 122.45, 115.78, 104.57, 103.98, 98.00, 82.38, 79.17, 73.84, 71.04, 70.93, 61.88.

Quercetin 8-*C*-glucoside ^1^H NMR (DMSO-*d*_*6*_, 400 MHz): *δ* 12.70 (s, 1H), 10.76 (s, 1H), 9.64 (s, 1H), 9.34 (s, 1H), 9.07 (s, 1H), 7.87 (s, 1H), 7.66 (d, *J* = 8.5 Hz, 1H), 6.85 (d, *J* = 8.5 Hz, 1H), 6.27 (s, 1H), 4.68 (d, *J* = 9.9 Hz, 2H), 4.09 – 3.52 (m, 6H), 3.26 (t, *J* = 9.1 Hz, 3H).

Quercetin 8-*C*-glucoside ^13^C NMR (DMSO-*d*_*6*_, 101 MHz): δ 176.52, 162.63, 160.11, 155.31, 148.06, 147.52, 145.45, 135.89, 122.79, 120.76, 116.36, 115.85, 104.48, 103.95, 97.94, 82.42, 79.23, 73.86, 71.02, 70.82, 61.93.

## Results and discussion

### Screening UDP-glucose biosynthesis pathways and E. coli hosts for C-glycosylflavonol production

**Fig. 1:**
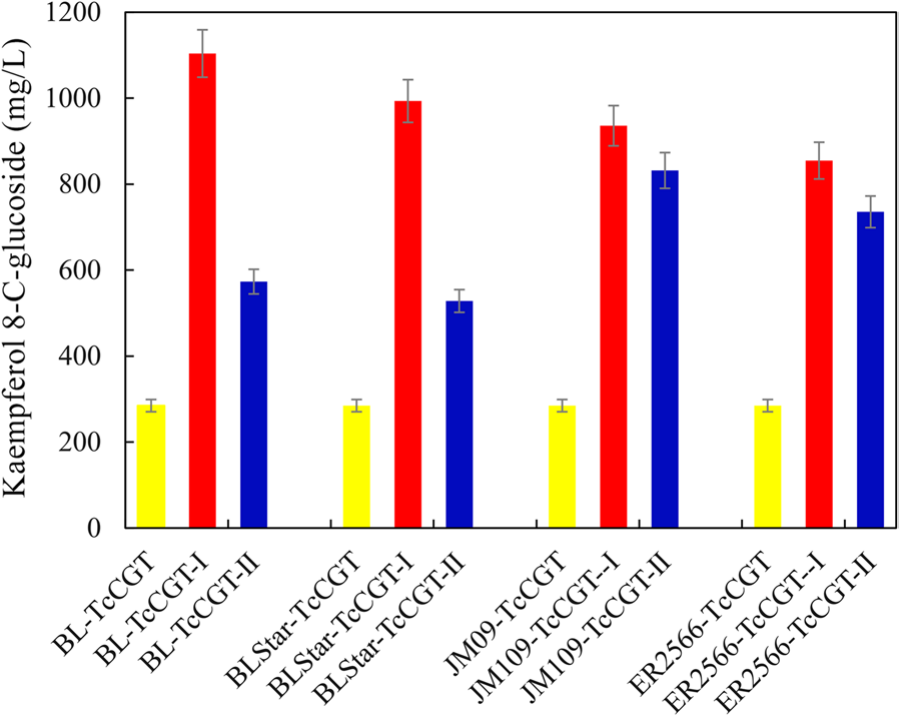
Effects of different UDP-glucose biosynthesis pathways and *E. coli* hosts on kaempferol 8-*C*-glucoside production. TcCGT represented hosts carrying pCDFDuet-TcCGT. TcCGT-I represented hosts carrying pCDFDuet-TcCGT and pACYCDuet-cscB-Cep-UgpA. TcCGT-II represented hosts carrying pCDFDuet-TcCGT and pACYCDuet-cscB-Basp-UgpA

Kaempferol 8-*C*-glucoside and quercetin 8-*C*-glucoside can be synthesized by *T. chinensis C*-glycosyltransferase (TcCGT) and transfer UDP-glucose to kaempferol and quercetin, respectively [32]. Thus, the catalytic capability of TcCGT and the supply of UDP-glucose are important factors for producing *C*-glycosylflavonols in the recombinant strains. The plasmids with two UDP-glucose biosynthesis pathways (the cellobiose phosphorolysis pathway and the sucrose phosphorolysis pathway) and the *TcCGT* gene were cotransformed into four *E. coli* strains (BL21 (DE3), BL21Star (DE3), JM109 (DE3), and ER2566) to determine whether different UDP-glucose biosynthesis pathways and strains would lead to differences in kaempferol 8-*C*-glucoside production (Fig. 1). The cellobiose phosphorolysis pathway begins with the reaction that phosphorylates cellobiose to produce glucose and glucose 1-phosphate by the action of cellobiose phosphorylase. Glucose 1-phosphate is then converted to UDP-glucose through the reaction catalyzed by UTP-glucose-1-phosphate uridylyltransferase (Additional file 1: Fig. S2a). In the sucrose phosphorolysis pathway, UDP-glucose is synthesized from UDP and sucrose through the consecutive two reactions by sucrose phosphorylase and UTP-glucose-1-phosphate uridylyltransferase (Additional file 1: Fig. S2b) [11, 35]. Kaempferol 8-*C*-glucoside production in BL21 (DE3) and BL21Star (DE3) harboring the cellobiose phosphorolysis pathway was higher than that in recombinant strains harboring the sucrose phosphorolysis pathway. However, there were no significant differences in kaempferol 8-*C*-glucoside production between JM109 (DE3) and ER2566 strains with the sucrose phosphorolysis pathway or the cellobiose phosphorolysis pathway. The maximum kaempferol 8-*C*-glucoside production in BL-TcCGT-I harboring the cellobiose phosphorolysis pathway was 1103 mg/L in TB-Gly medium for 24 h of bioconversion, which was approximately 193% of that in BL-TcCGT-II harboring the sucrose phosphorolysis pathway. The results indicated that BL21 (DE3) harboring the cellobiose phosphorolysis pathway represented a potential candidate.

### Optimization of the bioconversion conditions

**Fig. 2:**
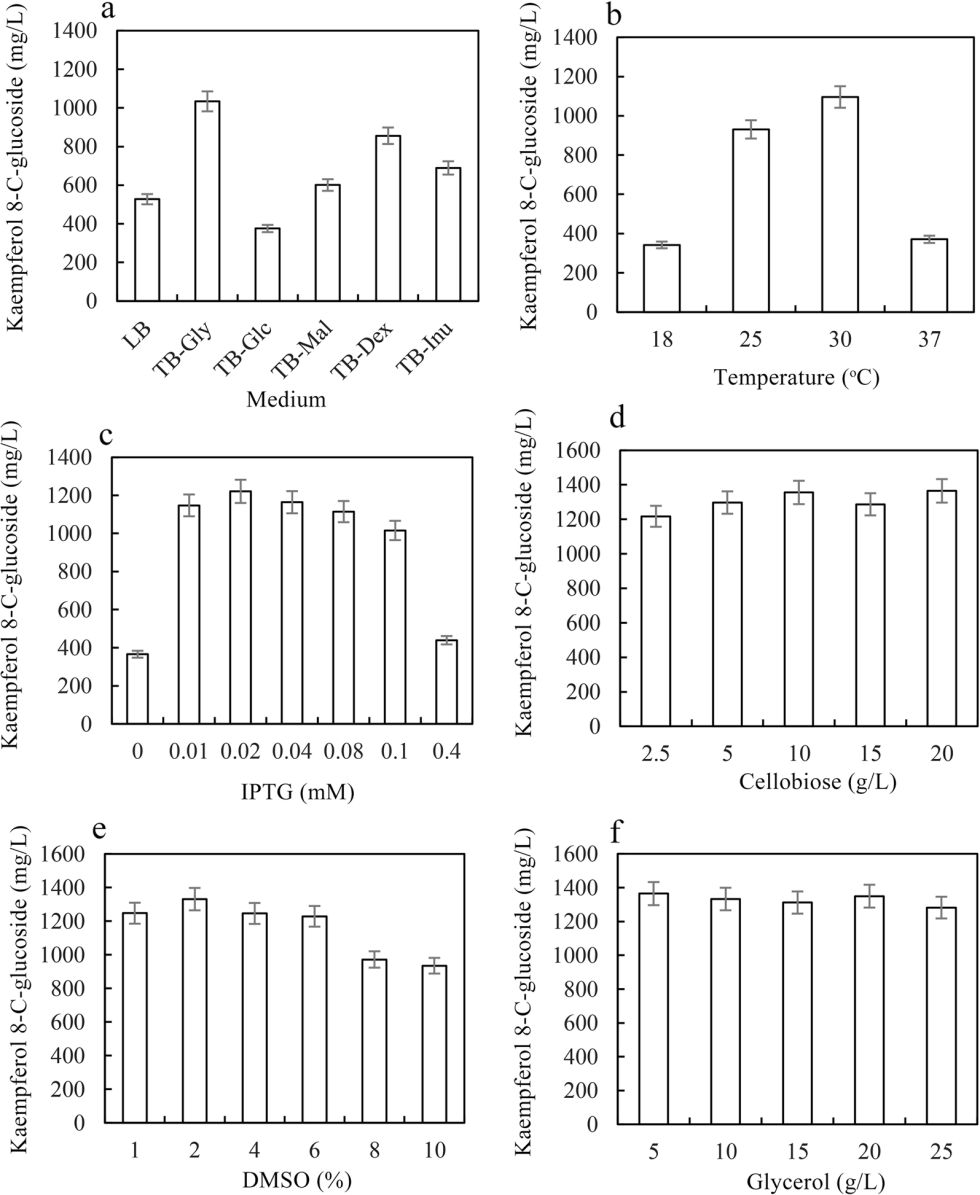
Optimization of the bioconversion conditions for kaempferol 8-*C*-glucoside production in BL-TcCGT-I. **a** Effects of different media on kaempferol 8-*C*-glucoside production. **b** Effects of temperature on kaempferol 8-*C*-glucoside production. **c** Effects of IPTG on kaempferol 8-*C*-glucoside production. **d** Effects of cellobiose on kaempferol 8-*C*-glucoside production. **e** Effects of DMSO on kaempferol 8-*C*-glucoside production. **f** Effects of glycerol on kaempferol 8-*C*-glucoside production. A total of 2 g/L kaempferol, 5 g/L cellobiose, and 0.1 mM IPTG were added to the recombinant strains, and the fermentation broth was incubated at 30 °C and 180 rpm for 24 h

*E. coli* can produce different amounts of acetic acid by metabolizing different carbon sources, while acetic acid can affect *C*-glycosylflavonol production by inhibiting the expression level of enzymes [36]. The effects of the medium with different carbon sources on kaempferol 8-*C*-glucoside production were determined. Among the six media, the highest kaempferol 8-*C*-glucoside production was 1034 mg/L for 24 h of bioconversion in TB-Gly, which was 275% of that observed in TB-Glc (Fig. 2a). The induction temperature and IPTG could affect the expression level of enzymes and the growth of recombinant strains. The optimal induction temperature for BL-TcCGT-I was 30 °C, and kaempferol 8-*C*-glucoside production reached 1095 mg/L for 24 h bioconversion (Fig. 2b). Kaempferol 8-*C*-glucoside production was 1220 mg/L after the addition of 0.02 mM IPTG, which was 333% of that without IPTG addition (Fig. 2c). The supply of UDP-glucose is closely related to cellobiose. The highest kaempferol 8-*C*-glucoside production reached 1355 mg/L with the addition of 10% cellobiose (Fig. 2d). Kaempferol 8-*C*-glucoside production was not affected when the DMSO concentration was less than or equal to 6%, but kaempferol 8-*C*-glucoside production decreased when the DMSO concentration exceeded 8% (Fig. 2e). There were no significant differences in kaempferol 8-*C*-glucoside production with increasing glycerol concentration (Fig. 2f).

**Fig. 3:**
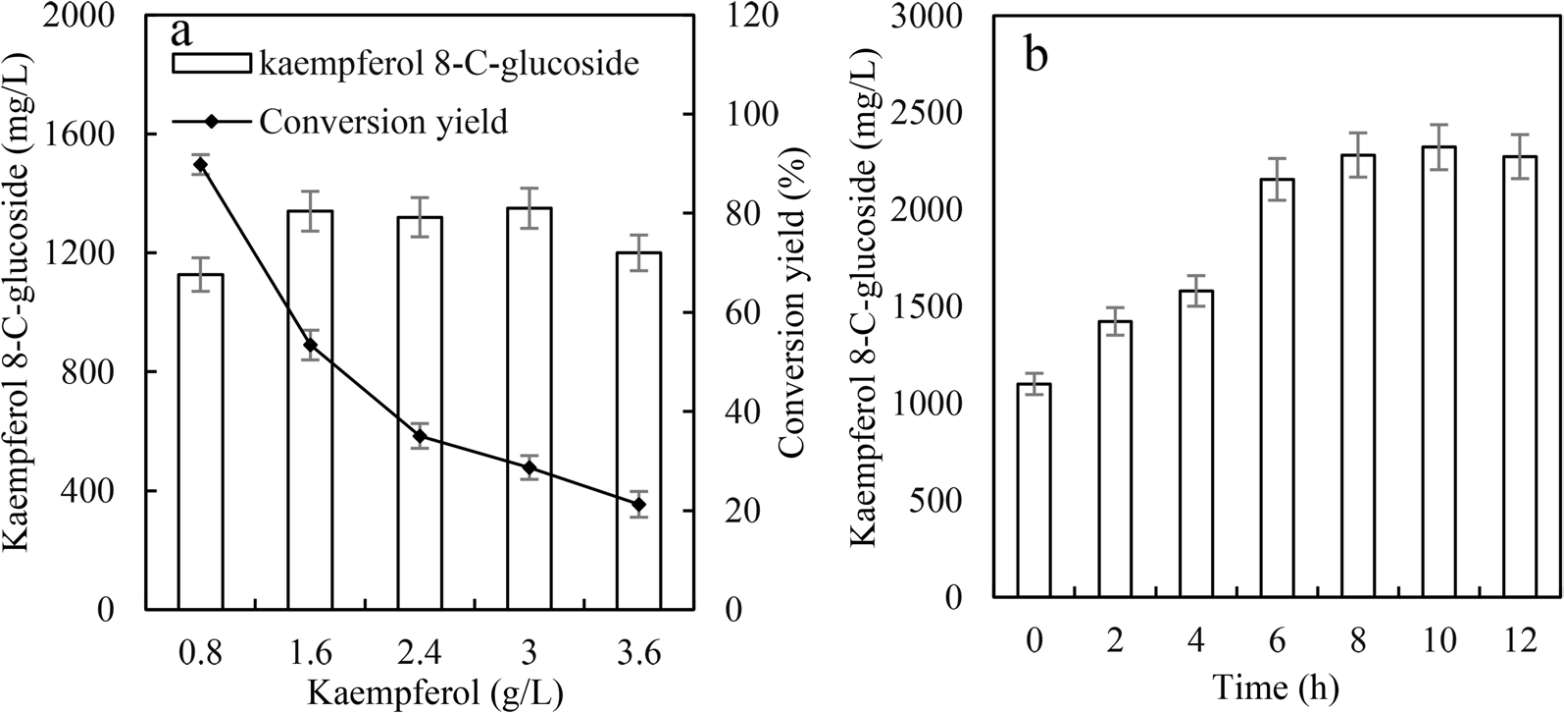
Effects of kaempferol and the timing of kaempferol addition on kaempferol 8-*C*-glucoside production. **a** Effects of kaempferol on kaempferol 8-*C*-glucoside production. A total of kaempferol (0.8, 1.6, 2.4, 3, and 3.6 g/L) was added to the recombinant strains. **b** Effects of the timing of kaempferol addition on kaempferol 8-*C*-glucoside production. 0.02 mM IPTG was added to the recombinant strains, and the recombinant strains were incubated at 20 °C and 180 rpm for (0, 2, 4, 6, 8, 10, and 12 h)

Flavonoids could affect the expression of recombinant proteins by inhibiting the growth of *E. coli*, which would result in decreased kaempferol 8-*C*-glucoside production [38, 39]. The effects of kaempferol and the timing of kaempferol addition were determined. Although kaempferol 8-*C*-glucoside production in BL-TcCGT-I did not decrease when the kaempferol concentration was less than 3 g/L, the conversion yield significantly decreased with increasing of kaempferol concentration (Fig. 3a). Kaempferol 8-*C*-glucoside production significantly increased when BL-TcCGT-I was first induced for a period of time to express recombinant enzymes, after which kaempferol was added to produce kaempferol 8-*C*-glucoside. The optimal timing was to perform the first induction for 10 h, followed by the addition of kaempferol. The highest kaempferol 8-*C*-glucoside production reached 2321 mg/L after 24 h of biotransformation, which was 211% of that of the control (Fig. 3b). These results indicate that kaempferol can affect kaempferol 8-*C*-glucoside production and that adjusting the timing of kaempferol addition can increase kaempferol 8-*C*-glucoside production.

### Effects of glycerol on avoiding degradation of C-glycosylflavonols

**Fig. 4:**
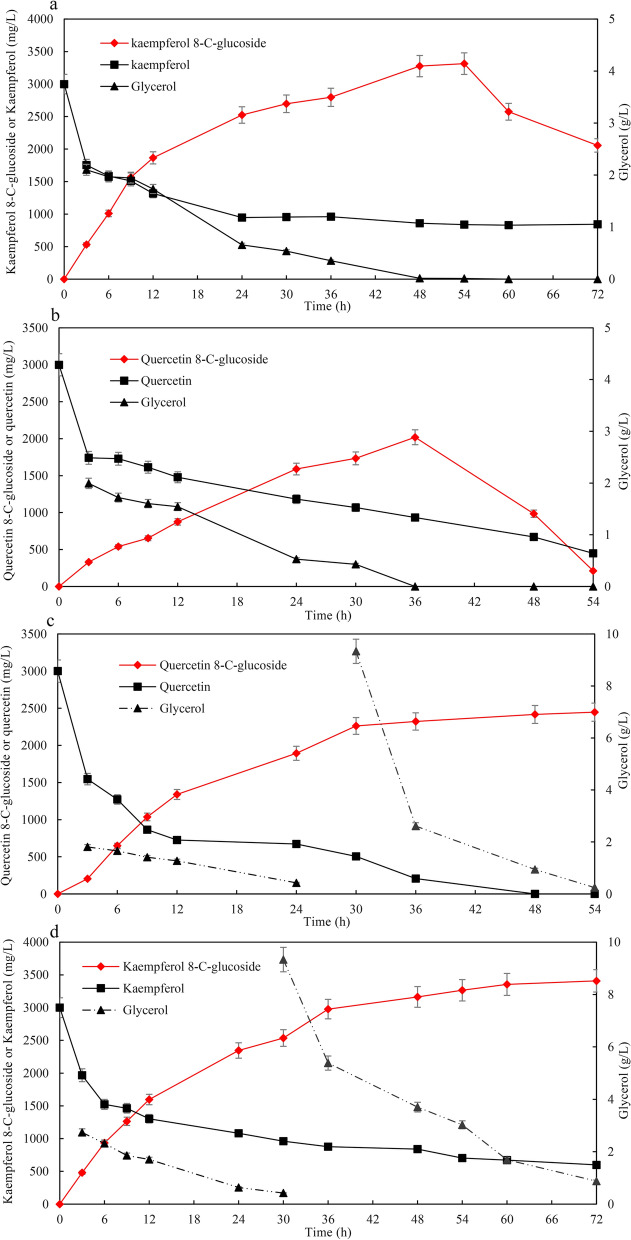
The time courses for flavonol consumption and *C*-glycosylflavonol production in BL-TcCGT-I. **a** The time courses for kaempferol consumption and kaempferol 8-*C*-glucoside production, **b** the time courses for quercetin consumption and quercetin 8-*C*-glucoside production, **c** the time courses for quercetin consumption and quercetin 8-*C*-glucoside production with the addition of glycerol. A total of 3 g/L kaempferol or quercetin, 10 g/L cellobiose, and 0.1 mM IPTG were added to the recombinant strains

Under the optimal bioconversion conditions, the time courses for flavonol consumption and *C*-glycosylflavonol production are given in Fig. 4. After 10 h of incubation and when 3000 mg/L kaempferol or quercetin was added to the medium, kaempferol 8-*C*-glucoside production was 3314 mg/L with a specific productivity of 61 mg/L/h after 54 h of biotransformation (Fig. 4a), and quercetin 8-*C*-glucoside production was 2019 mg/L with a specific productivity of 56 mg/L/h after 36 h of biotransformation (Fig. 4b). The specific productivity for kaempferol 8-*C*-glucoside during 0–12 h of bioconversion reached 155 mg/L/h, which was significantly higher than that during the whole process of biotransformation.

In the later stages of bioconversion, kaempferol 8-*C*-glucoside and quercetin 8-*C*-glucoside production decreased gradually due to degradation. Quercetin 8-*C*-glucoside production and the yield of substrate conversion were only 211 mg/L and 5.3%, respectively, after 54 h of biotransformation (Fig. 4b). Further study showed that *C*-glycosylflavonols began to degrade when glycerol was completely consumed (Fig. 4a, b). To improve *C*-glycosylflavonol production, fresh glycerol was added to the broths once it was consumed. Quercetin 8-*C*-glucoside production reached 2447 mg/L with the yield of substrate conversion of 52.4%, which was about 11 times higher than that of the control without adding fresh glycerol (Fig. 4c). Kaempferol 8-*C*-glucoside production reached 3408 mg/L after 72 h of biotransformation, which was 165% of the control without adding fresh glycerol (Fig. 4d). It has been reported that *E. coli* used the aromatic beta-glucosidases (arbutin, salicin, and esculin) as the carbon source [43]. These results suggested that adding glycerol could avoid the degradation of *C*-glycosylflavonols by probably suppressing catabolic systems for utilization of the aromatic beta-glucosidases. Although the quercetin 8-*C*-glucoside production and the yield of substrate conversion significantly increased with adding glycerol, about 47% quercetin was degraded during the fermentation process. The results indicated that adding glycerol could not avoid the degradation of quercetin. Thus, improving *C*-glycosylflavonol production and avoiding the degradation of substrate are key factors for the bioconversion.

### Resting cell bioconversion for C-glycosylflavonol production

**Fig. 5:**
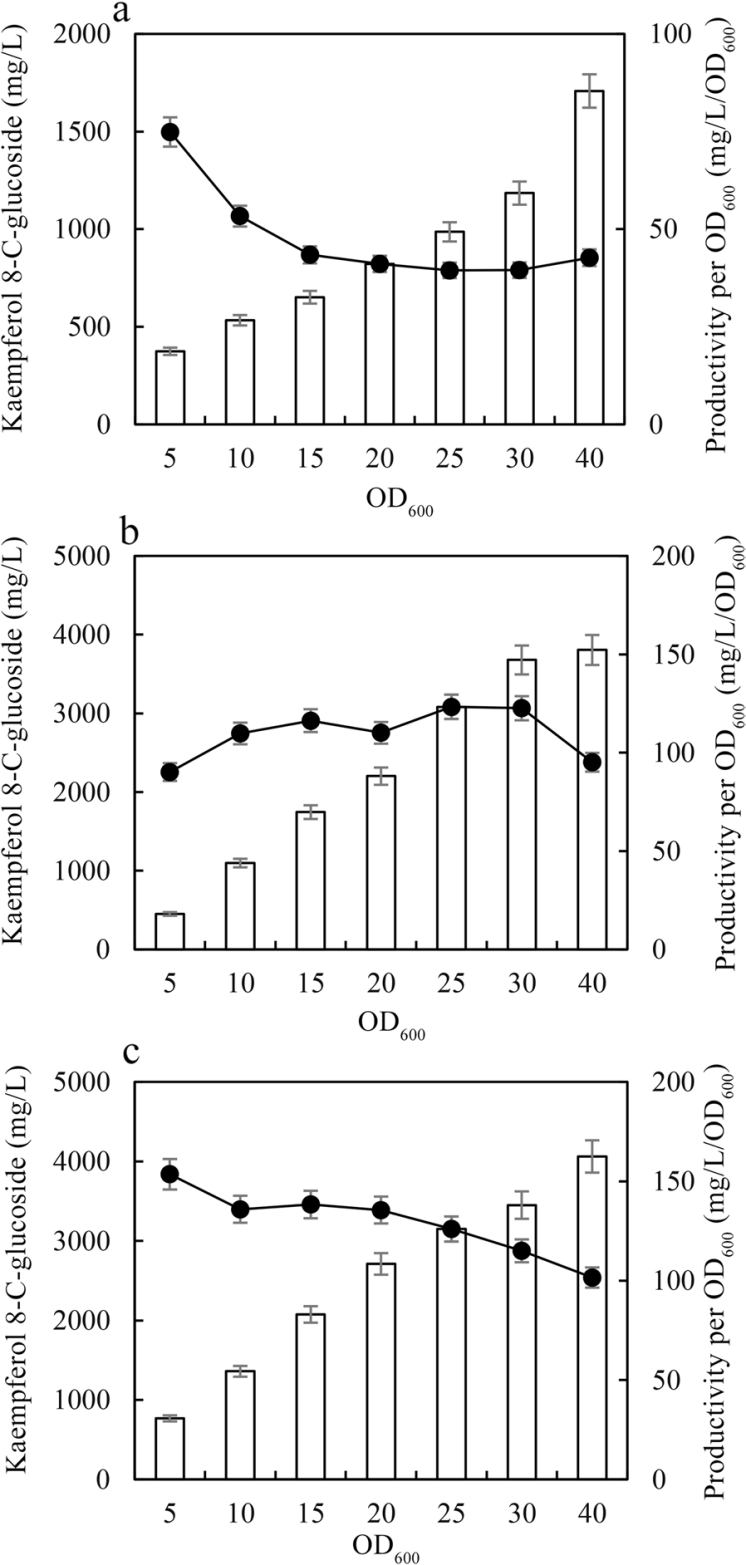
The effects of cell concentration on kaempferol 8-*C*-glucoside production with LB medium **a**, TB-Gly medium **b**, and M9-Gly medium **c**. Different concentrations of recombinant strains were incubated by using the broth (5 mL) in tubes at 30 °C for 24 h. A total of 3 g/L kaempferol or quercetin and 10 g/L cellobiose were added to the recombinant strains

Resting cell bioconversion has been used as an efficient method for the production of secondary metabolites, as it can improve production, avoid the degradations and inhibitions of substrates and products in vivo [40, 41]. The effects of cell concentration on kaempferol 8-*C*-glucoside production were determined (Fig. 5). Kaempferol 8-*C*-glucoside production improved with increasing resting cell concentration in LB, TB-Gly, and M9-Gly media, and the highest kaempferol 8-*C*-glucoside production was 4062 mg/L in M9-Gly medium (containing glycerol) with OD_600_ = 40 for 24 h bioconversion (Fig. 5). The productivity per OD_600_ decreased with increasing resting cell concentration in LB and M9-Gly medium, while the OD_600_ value was not significantly affected when the resting cell concentration OD_600_ was no more than 30 in TB-Gly medium (Fig. 5a–c). Kaempferol 8-*C*-glucoside production and the productivity per OD_600_ in M9-Gly and TB-Gly media were significantly higher than those in LB media. The main differences among them are that M9-Glyand TB-Gly media contained glycerol, while LB media are not supplemented with glycerol (Fig. 5).

**Fig. 6:**
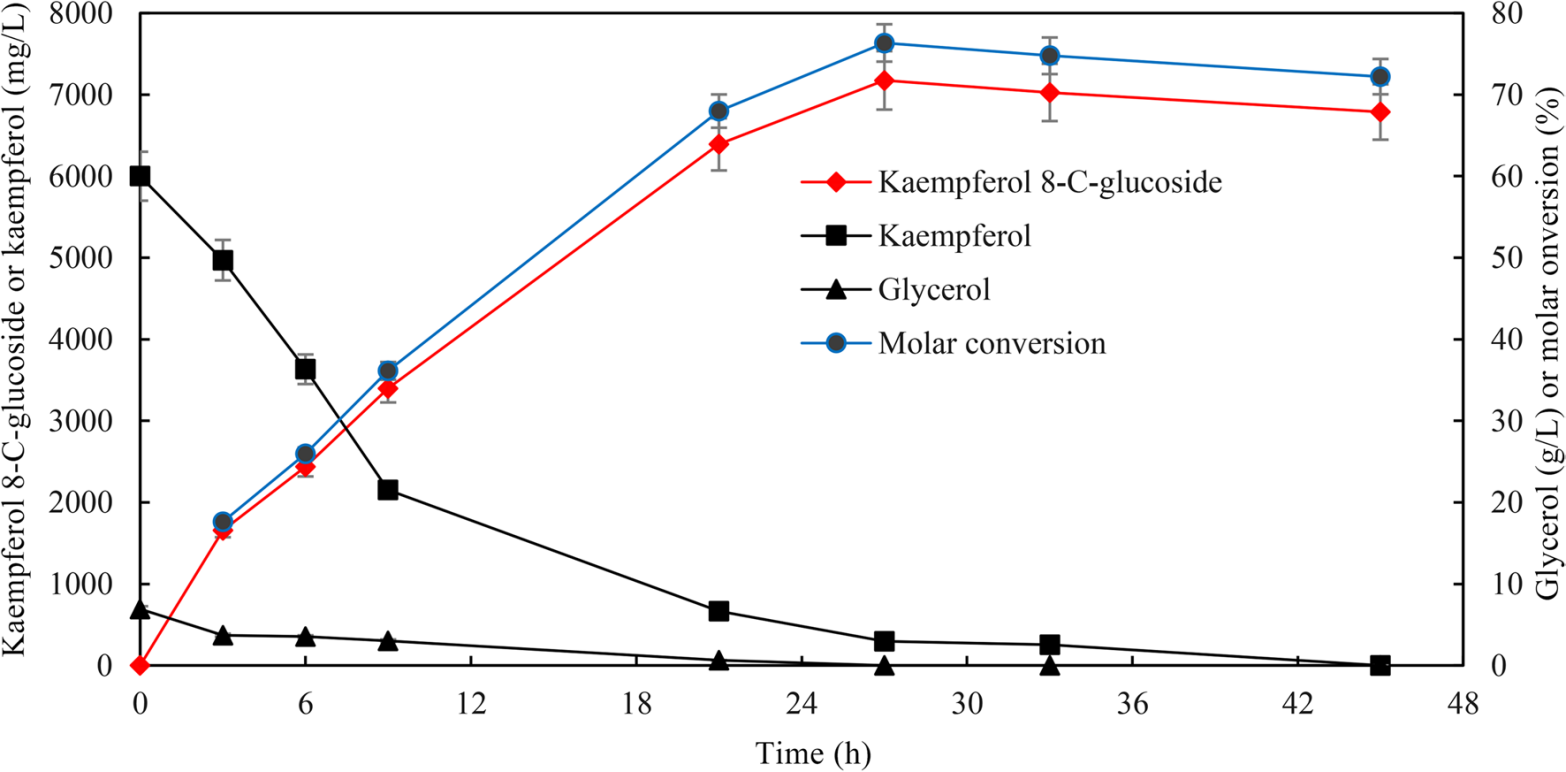
The time courses for kaempferol 8-*C*-glucoside production in M9-Gly medium. A total of 6 g/L kaempferol and 10 g/L cellobiose were added to the recombinant strains

The time courses for kaempferol 8-*C*-glucoside production in M9-Gly medium with OD_600_ = 40 are given in Fig. 6. Maximum kaempferol 8-*C*-glucoside production in M9-Gly medium reached 7027 mg/L after 33 h of bioconversion. The specific productivity reached 551 mg/L/h during 0–3 h of bioconversion, and the specific productivity remained at 263 mg/L/h during 3–21 h of bioconversion. At the end of bioconversion, kaempferol 8-*C*-glucoside began to degrade after 33 h of bioconversion because of the exhaustion of glycerol (Fig. 6).

**Fig. 7:**
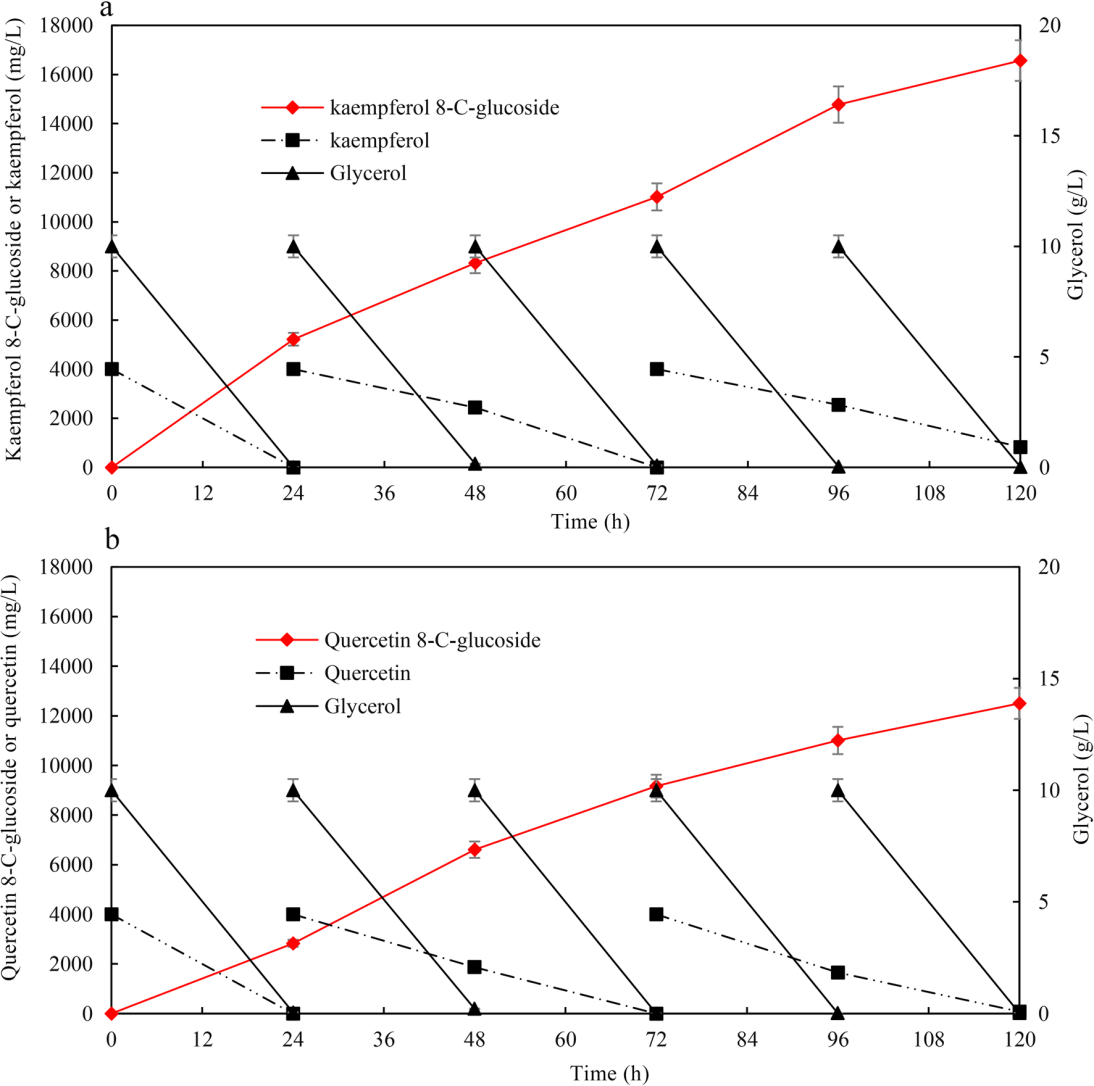
The time courses for *C*-glycosylflavonol production using fed-batch in M9-Gly medium. **a** The time courses for kaempferol 8-*C*-glucoside production by adding fresh substrates and glycerol, **b** the time courses for quercetin 8-*C*-glucoside production by adding fresh substrates and glycerol. In the fermentation process, 4 g/L kaempferol or quercetin and 10 g/L cellobiose were added to the broth at 24 h and 72 h, and 10 g/L glycerol was added to the broth at 24 h, 48 h, 72 h, and 96 h

To improve *C*-glycosylflavonol production and avoid the degradation of *C*-glycosylflavonols, we changed the bioconversion mode from batch to fed-batch by adding fresh substrate and glycerol once they had been consumed. According to the timing of substrate and glycerol addition, the process of bioconversion was divided into three parts as follows: 0–24, 24–72, and 72–120 h. At the beginning of bioconversion, the specific productivity of kaempferol 8-*C*-glucoside reached 217 mg/L/h during 0–24 h of bioconversion (Fig. 7a). With the increase in kaempferol 8-*C*-glucoside concentration, the specific productivity gradually decreased. The specific productivity of kaempferol 8-*C*-glucoside was 120 and 115 mg/L/h, respectively, during 24–72 and 72–120 h bioconversion. After 120 h of bioconversion, the maximal kaempferol 8-*C*-glucoside production was 16.6 g/L, with the yield of substrate conversion of 88.3% (Fig. 7a). HPLC analysis of kaempferol 8-*C*-glucoside production for 0, 48, and 120 h of incubation is shown in Additional file 1: Fig. S3. The specific productivity of quercetin 8-*C*-glucoside showed the same trend in the fed-batch bioconversion. The maximal quercetin 8-*C*-glucoside production was 12.5 g/L, and the specific productivity was 104 mg/L/h, with the yield of substrate conversion of 67.8% (Fig. 7b). Finally, about 32% quercetin was degraded during the resting cell bioconversion process, which was significantly lower than that (47%) of fermentation process. Thus, the degradation of the substrate decreased and the production of *C*-glycosylflavonols significantly improved by using the resting cell bioconversion and fed-batch. At the end of the bioconversion, there is only trace substrate in the bioconversion broth because of the bioconversion and degradation of the substrate in vivo. It will greatly reduce the difficulty of purification of kaempferol 8-*C*-glucoside and quercetin 8-*C*-glucoside. This study provides an efficient method for the biocatalytic synthesis of novel *C*-glycosylflavonols (kaempferol 8-*C*-glucoside and quercetin 8-*C*-glucoside) by the resting cell bioconversion. Although the production of *C*-glycosylflavonols was high by using resting cells biotransformation, the substrates, especially kaempferol are expensive. Kaempferol and quercetin can be prepared by the biotransformation of naringenin and naringenin was cheap [44]. Thus, *C*-glycosylflavonols can be biotransformed from naringenin by metabolic engineering of *Escherichia coli*.

### Screening macroporous resins for C-glycosylflavonol production

For the purification of *C*-glycosylflavonols with macroporous resins, the adsorption/desorption capacity is the most important indicator [45, 46]. A total of 6 types of macroporous resins, including one strong polar (NKA-9), one semipolar (DM 301), two weak polar (AB-8), and three nonpolar (D101, HPD100, and HP20), were determined to evaluate their adsorption/desorption capacity for kaempferol 8-*C*-glucoside. The adsorption capacity (22.28 mg/g), desorption capacity (17.04 mg/g) and recovery (55.71%) for kaempferol 8-*C*-glucoside on HPD100 were higher than those on other macroporous resins (Table 2). Therefore, HPD100 was selected as a potential candidate for the adsorption/desorption of *C*-glycosylflavonols.

**Table 2:** Adsorption/desorption capacity and recovery for kaempferol 8-C-glucoside using different macroporous resins

Type	Polarity	Adsorption capacity (mg/g)	Adsorption ratio (%)	Desorption capacity (mg/g)	Desorption ratio (%)	Recovery (%)
NKA-9	Strong-polar	17.33 ± 0.35	56.65 ± 1.13	11.14 ± 0.02	64.27 ± 1.84	36.41 ± 0.47
DM301	Semipolar	15.59 ± 0.16	50.95 ± 0.41	10.21 ± 0.32	65.54 ± 0.77	33.39 ± 0.98
AB-8	Weak-polar	22.2 ± 0.22	72.57 ± 0.65	16.37 ± 0.21	73.73 ± 0.57	53.50 ± 0.55
D101	Nonpolar	19.03 ± 0.76	62.2 ± 1.24	13.71 ± 0.15	72.07 ± 0.78	44.82 ± 0.54
HPD100	Nonpolar	22.28 ± 0.44	72.83 ± 0.51	17.04 ± 0.44	76.49 ± 0.39	55.71 ± 0.21
HP-20	Nonpolar	21.71 ± 0.17	70.98 ± 0.71	16.10 ± 0.42	74.15 ± 0.80	52.62 ± 0.77

Data are represented as mean ± SD of three independent experiments

### Adsorption and desorption kinetics

**Fig. 8:**
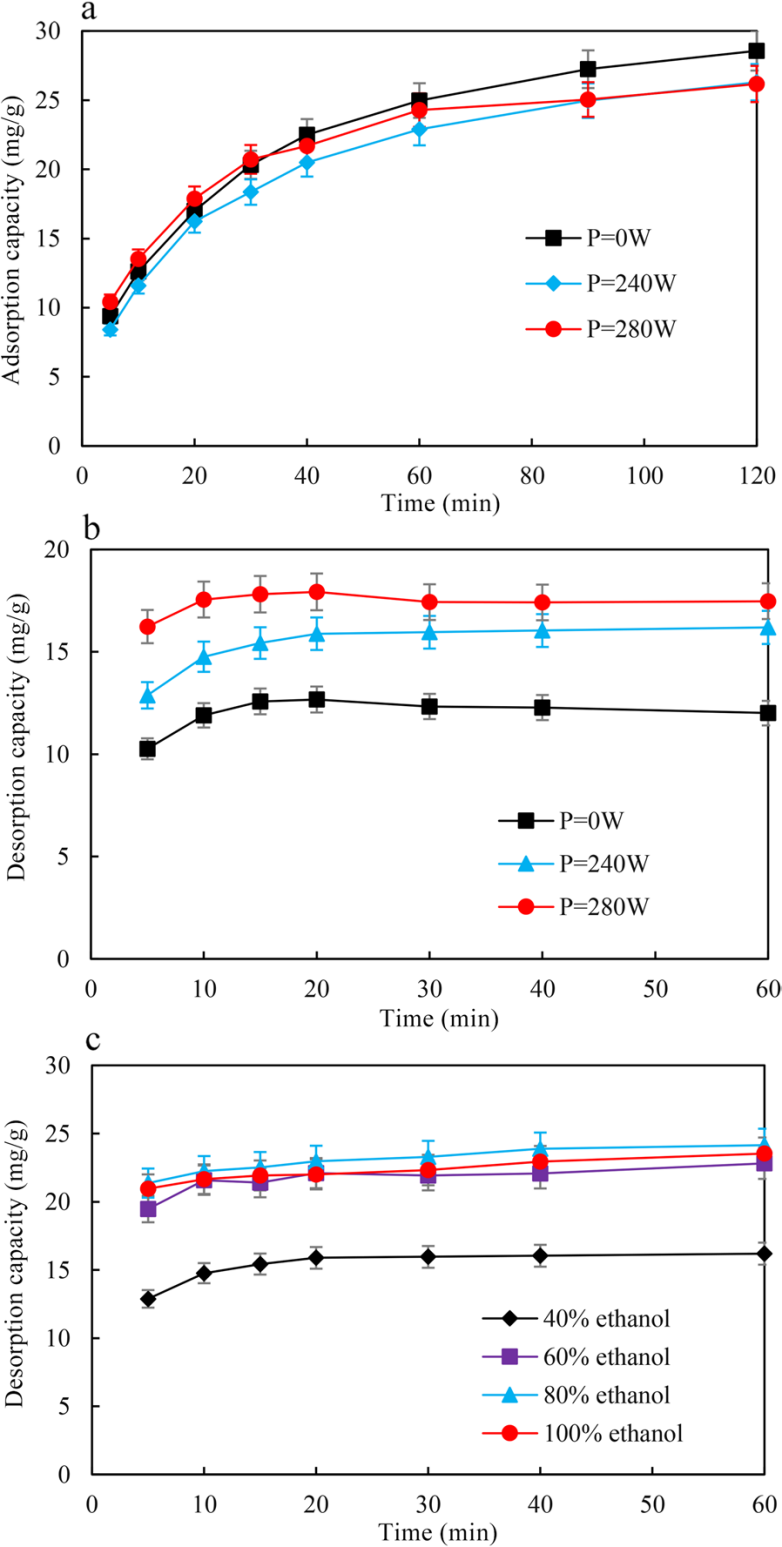
Absorption kinetics and desorption of kaempferol 8-*C*-glucoside on HPD100 under different conditions. **a** Absorption kinetics of kaempferol 8-*C*-glucoside on HPD100 under different ultrasound powers, **b** desorption of kaempferol 8-*C*-glucoside on HPD100 under different ultrasound powers with 40% ethanol, **c** desorption of kaempferol 8-*C*-glucoside on HPD100 under ultrasound power (240 W) with different ethanol concentrations

The effects of ultrasound treatments on adsorption kinetics are shown in Fig. 8. At the beginning of adsorption, the kaempferol 8-*C*-glucoside adsorption capacity on HPD100 increased rapidly and then increased slowly until equilibrium (Fig. 8a). To analyze the adsorption kinetics of kaempferol 8-*C*-glucoside on HPD100 resin, pseudofirst-order and pseudosecond-order models were carried out under different ultrasound powers at 25 °C (Table 3). However, the adsorption capacity of kaempferol 8-*C*-glucoside did not show a significant difference under the different ultrasound powers. Thus, the optimal adsorption conditions were shaking at 25 °C and 80 rpm for 120 min, and the highest adsorption capacity reached 28.57 mg/g (Fig. 8a).

**Table 3:** Kinetic parameters of adsorption of kaempferol 8-C-glucoside using HPD100

Treatment type	Pseudofirst-order model R_1_^2^	Pseudosecond-order model				Particle diffusion kinetics model			
		R_2_^2^	Equation	K_1_ (g/mg min^−1^)	Qe (mg/g dry resin)	R_3_^2^	Equation	K_2_ (mg/g.min^1/2^)	M (mg/g)
0 W	0.9986	0.9979	t/Q_t_ = 0.0313 t + 0.4927	1.988 × 10^–3^	31.95	0.9432	Q_t_ = 2.2081 t^1/2^ + 6.5642	2.2081	6.5642
240 W	0.9978	0.9980	t/Q_t_ = 0.0340 t + 0.4384	2.637 × 10^–3^	29.41	0.9429	Q_t_ = 2.0269 t^1/2^ + 6.0230	2.0269	6.0230
280 W	0.9758	0.9991	t/Q_t_ = 0.0354 t + 0.3778	3.317 × 10^–3^	28.25	0.8981	Q_t_ = 1.7668 t^1/2^ + 8.9557	1.7668	8.9557

The effects of different ultrasound powers (*P* = 0, 240 and 280 W) and ethanol concentrations (40%–100%, v/v) on desorption kinetics are shown in Fig. 8b,c. The results revealed that the desorption capacity of kaempferol 8-*C*-glucoside on HPD100 increased rapidly with ultrasound power and reached equilibrium in less than 30 min (Fig. 8b). The desorption capacity of kaempferol 8-*C*-glucoside on HPD100 with ultrasound power (240 W or 280 W) was higher than that without ultrasound power (Fig. 8b). The high energy level of ultrasound treatment may destroy the surface structure of the macroporous resins, so the ultrasound power *P* = 240 W was used for subsequent desorption experiments. Ethanol is commonly used as a desorbent for macroporous resins because of its low toxicity, low cost, and easy removal [47, 48]. Thus, the desorption capacity on HPD100 was determined in different concentrations of ethanol. The highest desorption capacity reached 24.15 mg/g in 80% ethanol, and the desorption capacity decreased when the ethanol concentration reached 100% (Fig. 8c). Thus, the optimal desorption conditions were an ultrasound power of 240 W, an ethanol concentration of 80%, and a temperature of 25 °C for 60 min, and the highest desorption capacity reached 24.15 mg/g (Fig. 8b, c).

### Preparation and structural identification of C-glycosylflavonols

**Fig. 9:**
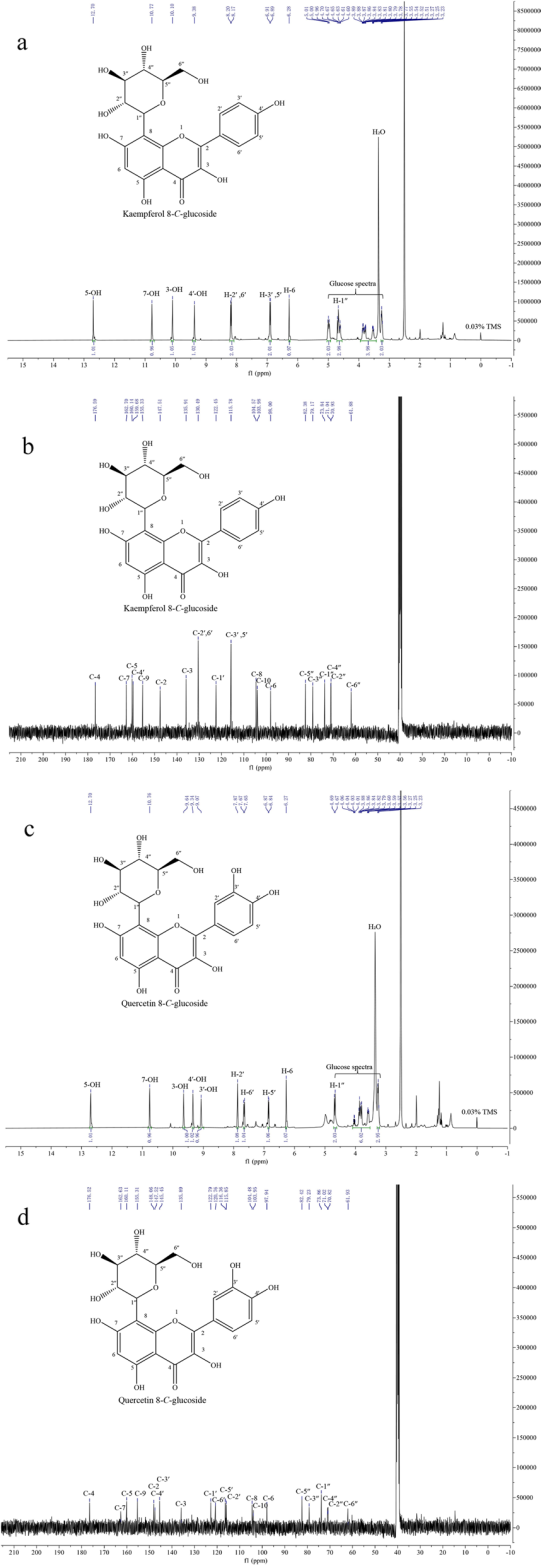
^1^H-NMR and ^13^C-NMR spectra of kaempferol 8-C-glucoside and quercetin 8-C-glucoside (400 MHz for ^1^H-NMR and 101 MHz for ^13^C-NMR, DMSO-*d*_*6*_, *δ* in ppm). **a** ^1^H-NMR for kaempferol 8-C-glucoside, **b** ^13^C-NMR for kaempferol 8-C-glucoside, **c** ^1^H-NMR for quercetin 8-C-glucoside, **d** ^13^C-NMR for quercetin 8-C-glucoside

According to the abovementioned adsorption and desorption kinetics results, a total of 1 L of resting cell bioconversion broth was used to prepare *C*-glycosylflavonols. Finally, kaempferol 8-*C*-glucoside (15.4 g) and quercetin 8-*C*-glucoside (11.3 g) were obtained with 93% and 91% yields, respectively. As shown in Additional file 1: Fig. S4, two new peaks (retention times of 4.084 and 3.683 min) were present in the conversion broth in BL-TcCGT-I with kaempferol and quercetin as the substrates (Additional file 1: Fig. S4b, d). A comparison of the m/z values of the molecular ions [M–H]^–^ of the bioconversion product (447.0957) showed that differences corresponded to a D-glucose residue of kaempferol (285.0417 Additional file 1: Fig. S5), and the experiment with quercetin as the substrate showed the same result (Additional file 1: Fig. S5). Moreover, the ^1^H NMR and ^13^C NMR spectra of the bioconversion products were analyzed (Fig. 9). In the ^1^H NMR in DMSO-*d*_6,_ a ^1^H singlet at *δ* 6.28 and 6.27 ppm were assigned as H-6, and H-8 signal was not detected in ^1^H NMR spectrum (Fig. 9a, c). Besides, ^13^C NMR spectrum shows peaks for *C*-6 and *C*-8 carbons at *δ* 98.00 and 104.57, respectively (Fig. 9b, d), which is different with previous studies on flavonol 6-*C*-glucoside [25]. These results confirmed that the bioconversion products were kaempferol 8-*C*-glucoside and quercetin 8-*C*-glucoside (Fig. 9).

## Conclusions

IN this study, a recombinant strain was reconstructed by screening UDP-glucose biosynthesis pathways and *E. coli* hosts to biosynthesize kaempferol 8-*C*-glucoside and quercetin 8-*C*-glucoside from kaempferol and quercetin, respectively. By adjusting the timing of flavonol addition, adding glycerol, and adopting resting cell bioconversion, the highest kaempferol 8-*C*-glucoside and quercetin 8-*C*-glucoside production reached 16.6 g/L and 12.5 g/L, respectively. By screening macroporous resins, the adsorption/desorption capacity of the nonpolar HPD100 showed the best features. The optimal adsorption conditions were 25 °C and 80 rpm for 120 min, with the highest adsorption capacity of 28.57 mg/g. The optimal desorption conditions were an ultrasound power of 240 W, an ethanol concentration of 80%, and a temperature of 25 °C for 60 min, and the highest desorption capacity reached 24.15 mg/g. Finally, kaempferol 8-*C*-glucoside (15.4 g) with a yield of 93% and quercetin 8-*C*-glucoside (11.3 g) with a yield of 91% were obtained from 1 L of fermentation broth. This efficient method provides a widely available approach for preparing and biosynthesizing *C*-glycosylflavonols via an environmentally safe process.

## Supplementary Information

**Additional file 1: Table S1.** Physical properties of different macroporous resins used in this study. **Figure S1.** The diagrams for the adsorption and desorption of C-glycosylflavonols via the macroporous resins. **Figure S2**. Biosynthesis of UDP-glucose by the cellobiose phosphorolysis pathway and sucrose phosphorolysis pathway. **Figure S3.** HPLC analysis of kaempferol 8-C-glucoside production for 0, 48, and 120 h of incubation and purified kaempferol 8-C-glucoside. **Figure S4.** HPLC analysis of kaempferol 8-C-glucoside and quercetin 8-C-glucoside production in BL-TcCGT-I. **Figure S5.** Liquid chromatography–mass spectrometry (LC/MS) analyses of the product formed from kaempferol and quercetin.

## Data Availability

All data generated or analyzed during this study are included in this published article and its supplementary information files.
