# Supplementary material for: An efficient preparation and biocatalytic synthesis of novel C-glycosylflavonols kaempferol 8-C-glucoside and quercetin 8-C-glucoside through using resting cells and macroporous resins
Source: Biotechnol Biofuels Bioprod. 2022 Nov 24;15:129. doi: 10.1186/s13068-022-02228-5 (PMC9700910; doi:10.1186/s13068-022-02228-5)
Supplement: Supplementary file 1 — Additional file 1: Table S1. Physical properties of different macroporous resins used in this study. Figure S1. The diagrams for the adsorption and desorption of C-glycosylflavonols via the macroporous resins. Figure S2. Biosynthesis of UDP-glucose by the cellobiose phosphorolysis pathway and sucrose phosphorolysis pathway. Figure S3. HPLC analysis of kaempferol 8-C-glucoside production for 0, 48, and 120 h of incubation and purified kaempferol 8-C-glucoside. Figure S4. HPLC analysis of kaempferol 8-C-glucoside and quercetin 8-C-glucoside production in BL-TcCGT-I. Figure S5. Liquid chromatography–mass spectrometry (LC/MS) analyses of the product formed from kaempferol and quercetin. [file 13068_2022_2228_MOESM1_ESM.docx]

**An efficient preparation and biocatalytic synthesis of novel *C*-glycosylflavonols kaempferol 8-*C*-glucoside and quercetin 8-*C*-glucoside through using resting cells and macroporous resins**

Yangbao Wu^1,2^, Huan Wang^1,2^, Yang Liu^1,2^, Linguo Zhao^1,2*^, Jianjun Pei^1,2^[[1]](#footnote-1)^*^

*^1^ Jiangsu Co-Innovation Center of Efficient Processing and Utilization of Forest Resources, College of Chemical Engineering, Nanjing Forestry University, Nanjing 210037, China*

*^2^ Jiangsu Key Lab of Biomass-Based Green Fuels and Chemicals, Nanjing 210037, China*

**SUPPORTING INFORMATION**

**Table S1** Physical properties of different macroporous resins used in this study.

| Type | Polarity | Particle  (mm) | Surface Area(m^2^/g) | Average Pore Diameter (nm) | Water content (%) |
| --- | --- | --- | --- | --- | --- |
| NKA-9 | Strong polar | 0.3-1.25 | 500-550 | 10-12 | 65-75 |
| DM301 | Semi-polar | 0.3-1.25 | 500-550 | 10-12 | 65-75 |
| AB-8 | Weak-polar | 0.3-1.25 | 480-520 | 12-14 | 65-75 |
| D101 | Non-polar | 0.3-1.25 | 550-600 | 9-10 | 65-75 |
| HPD100 | Non-polar | 0.3-1.20 | 650-700 | 9-10 | 65-75 |
| HP-20 | Non-polar | 0.3-1.25 | 550-600 | 10-12 | 65-75 |


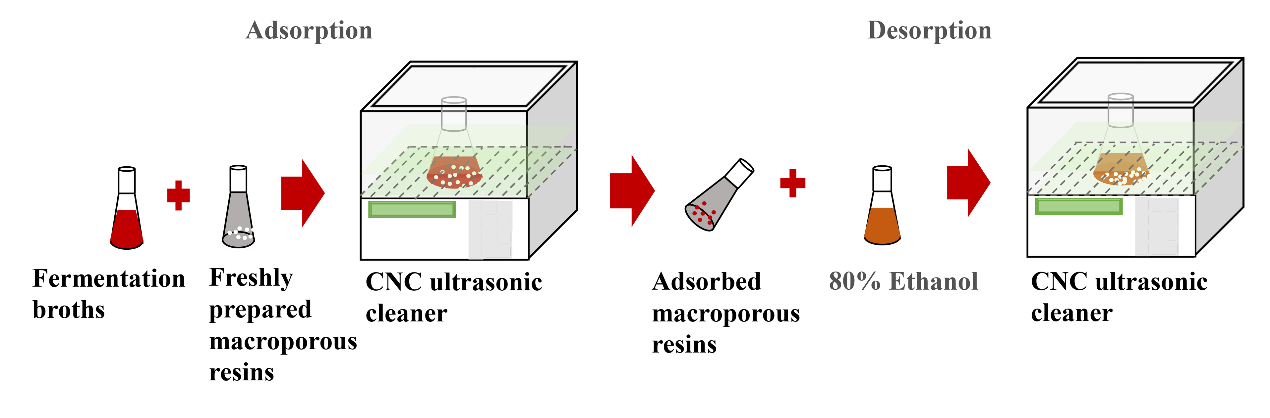


**Figure S1.** The diagrams for the adsorption and desorption of C-glycosylflavonols via the macroporous resins.


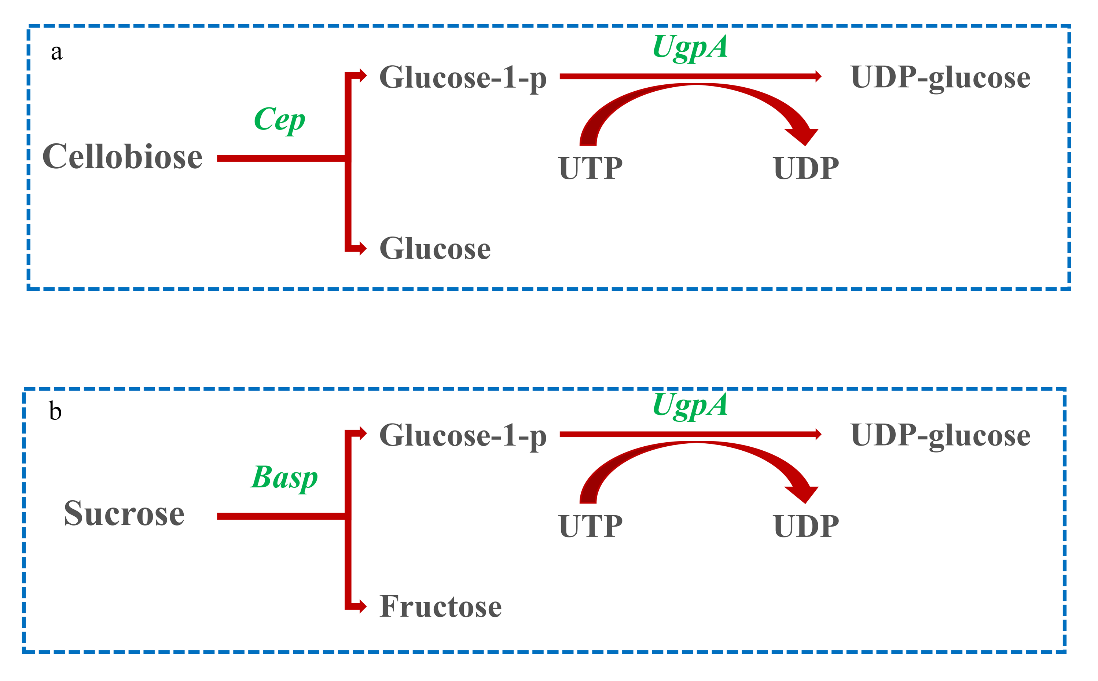


**Figure S2.** Biosynthesis of UDP-glucose by the cellobiose phosphorolysis pathway (a) and sucrose phosphorolysis pathway (d). Cep: cellobiose phosphorylase, Basp: sucrose phosphorylase, UgpA: uridylyltransferase.


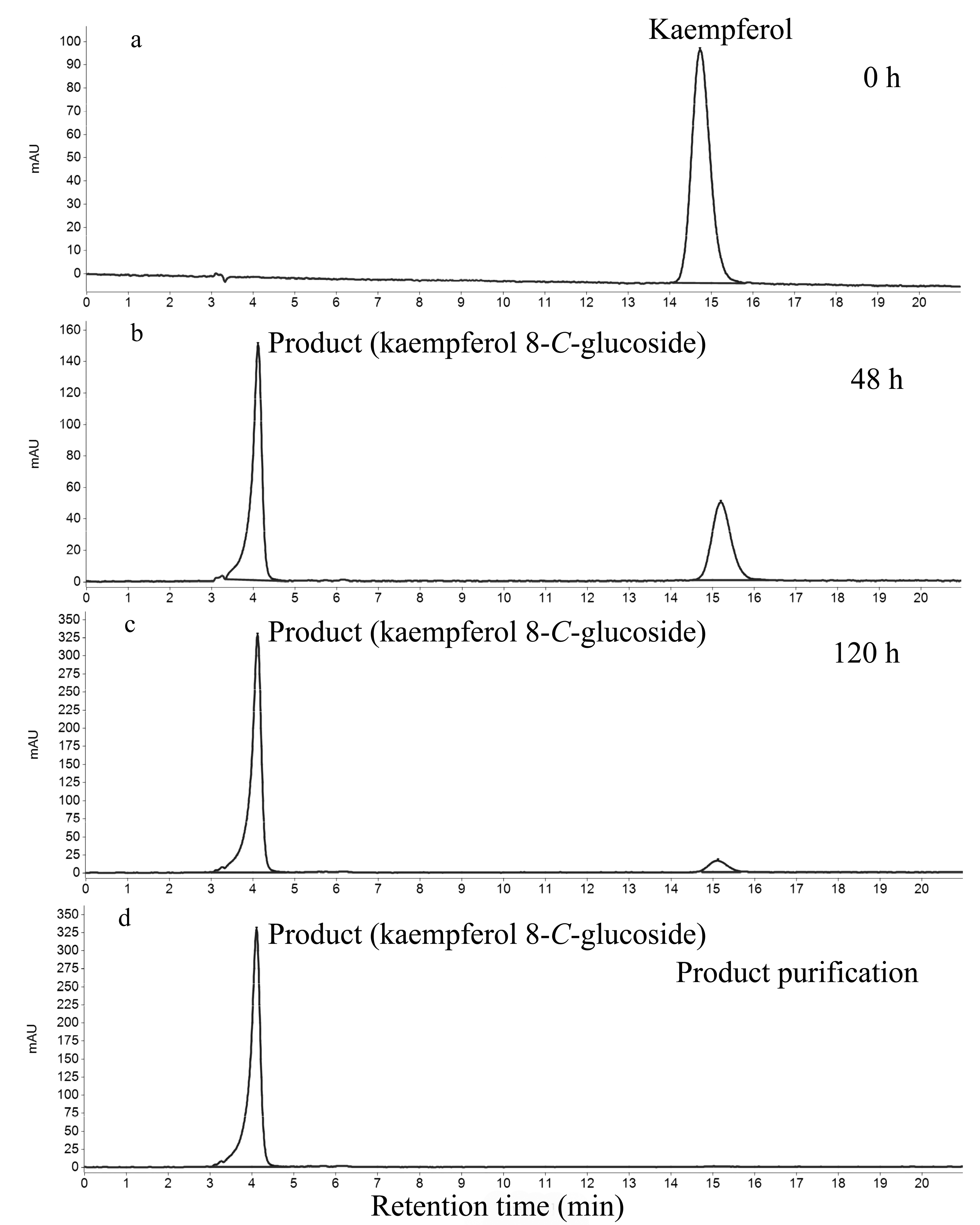


**Figure S3.** HPLC analysis of kaempferol 8-C-glucoside production for 0 (a), 48 (b), and 120 h (c) of incubation and purified kaempferol 8-C-glucoside (d).


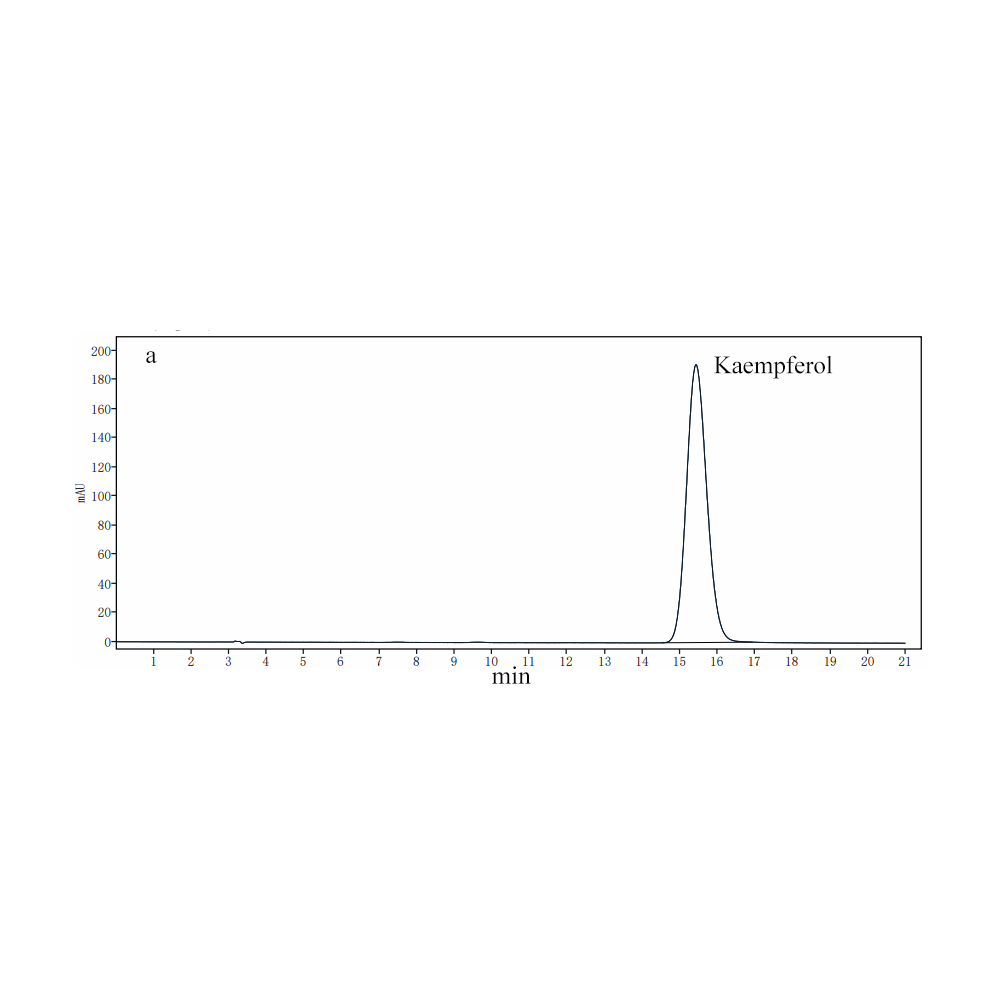


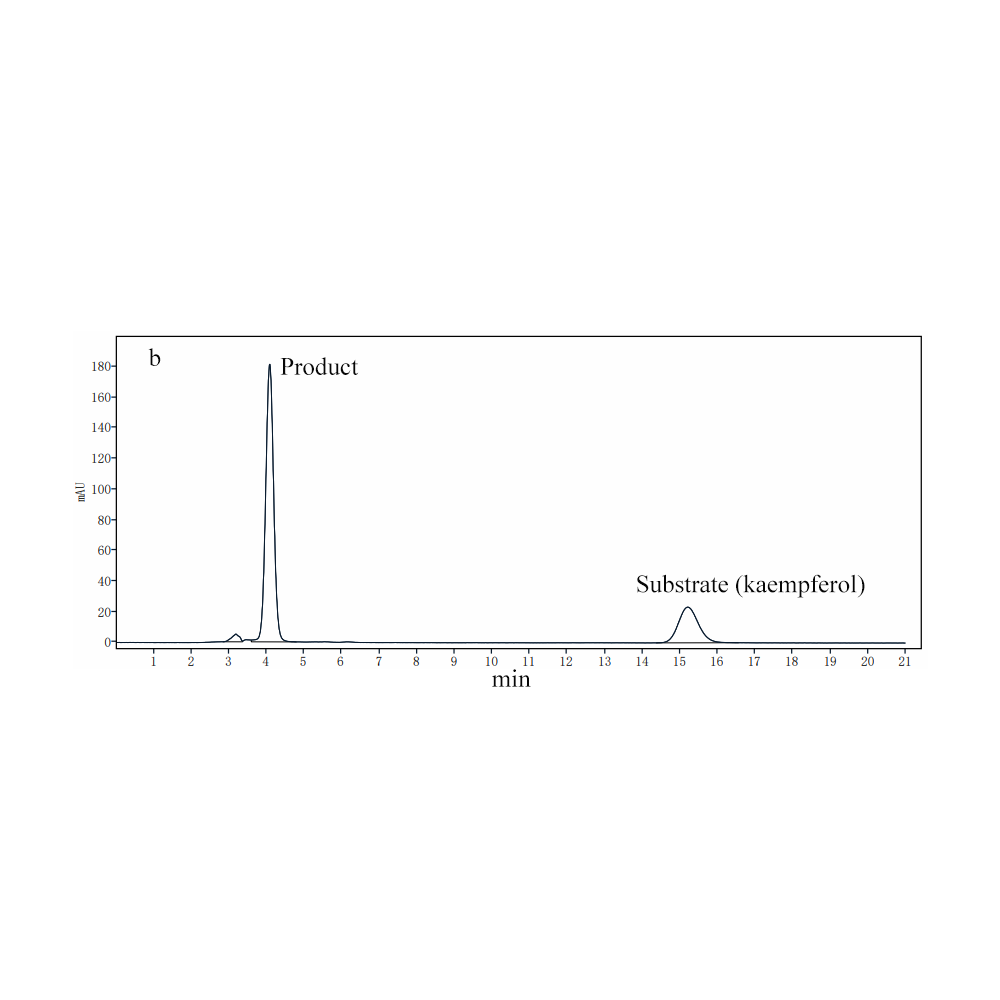


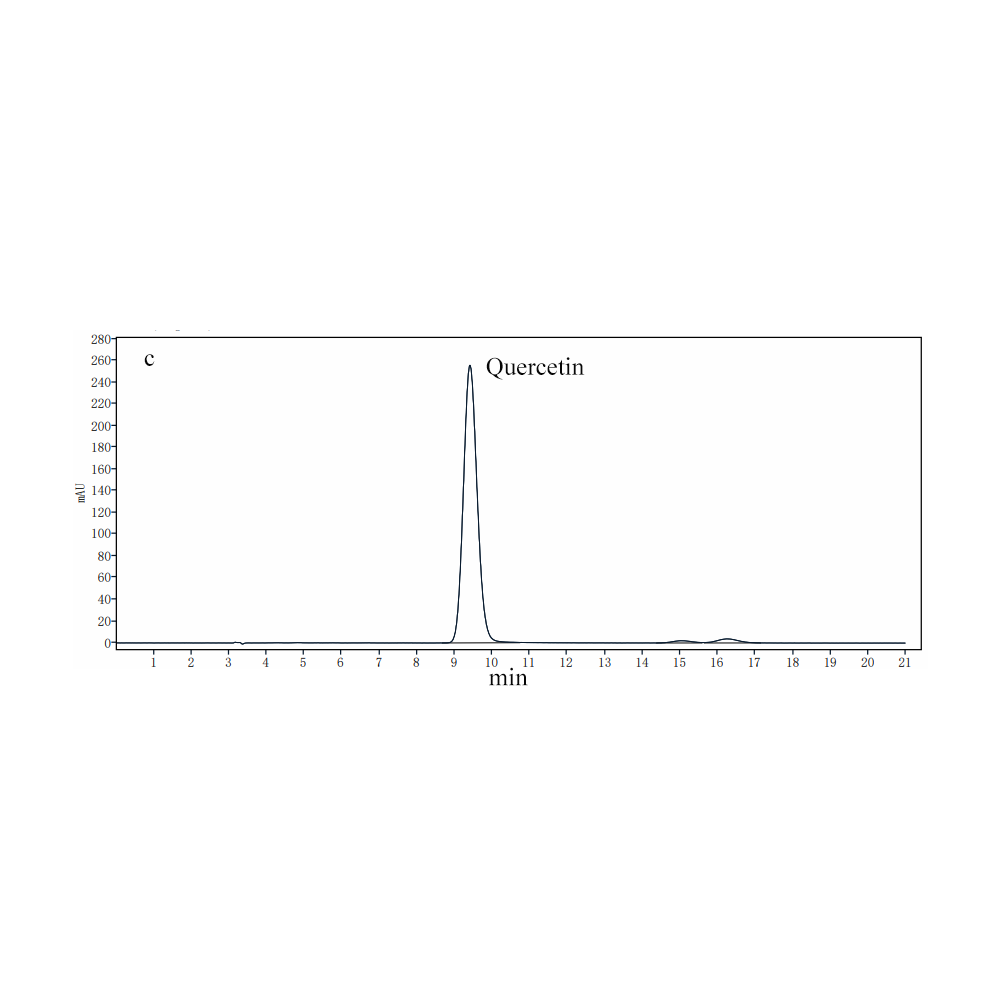


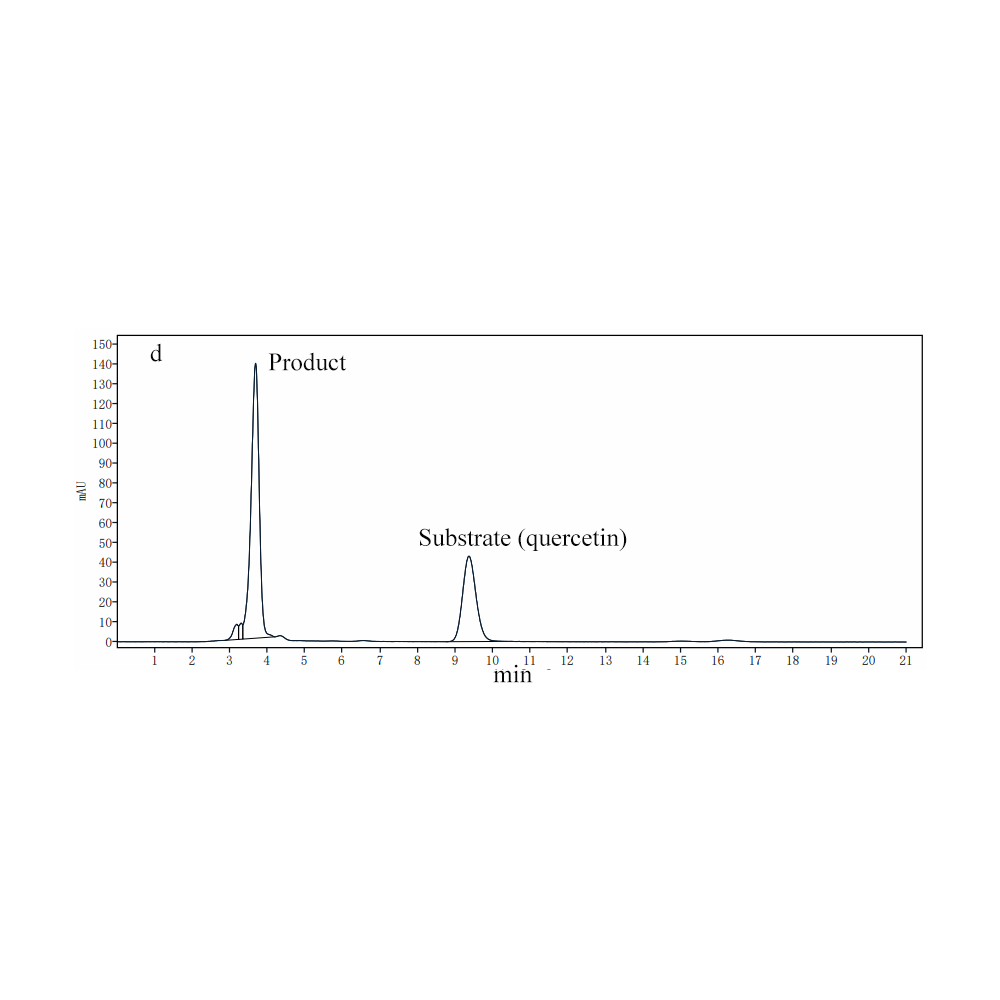


**Figure S4.** HPLC analysis of kaempferol 8-C-glucoside and quercetin 8-C-glucoside production in BL-TcCGT-I. (a) authentic kaempferol, (b) fermentation broth with kaempferol as substrate in BL-TcCGT-I, (c) authentic quercetin, (d) fermentation broth with quercetin as substrate in BL-TcCGT-I.

**
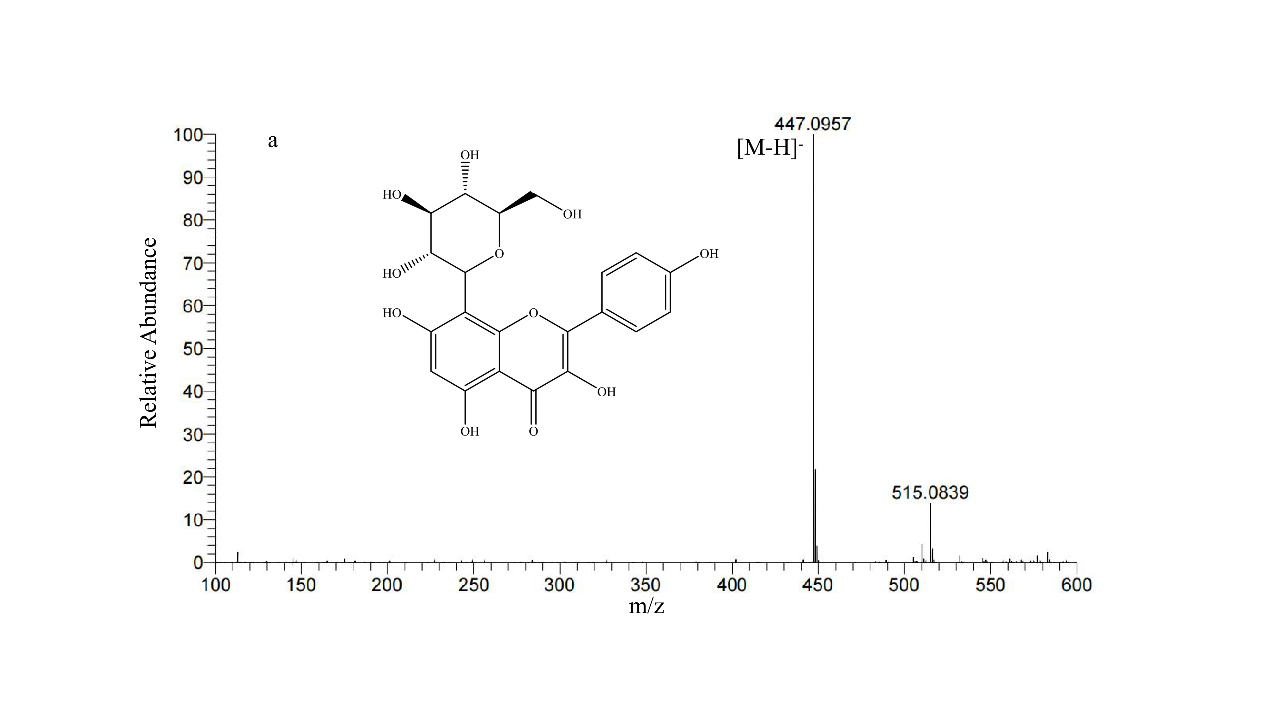
**

**
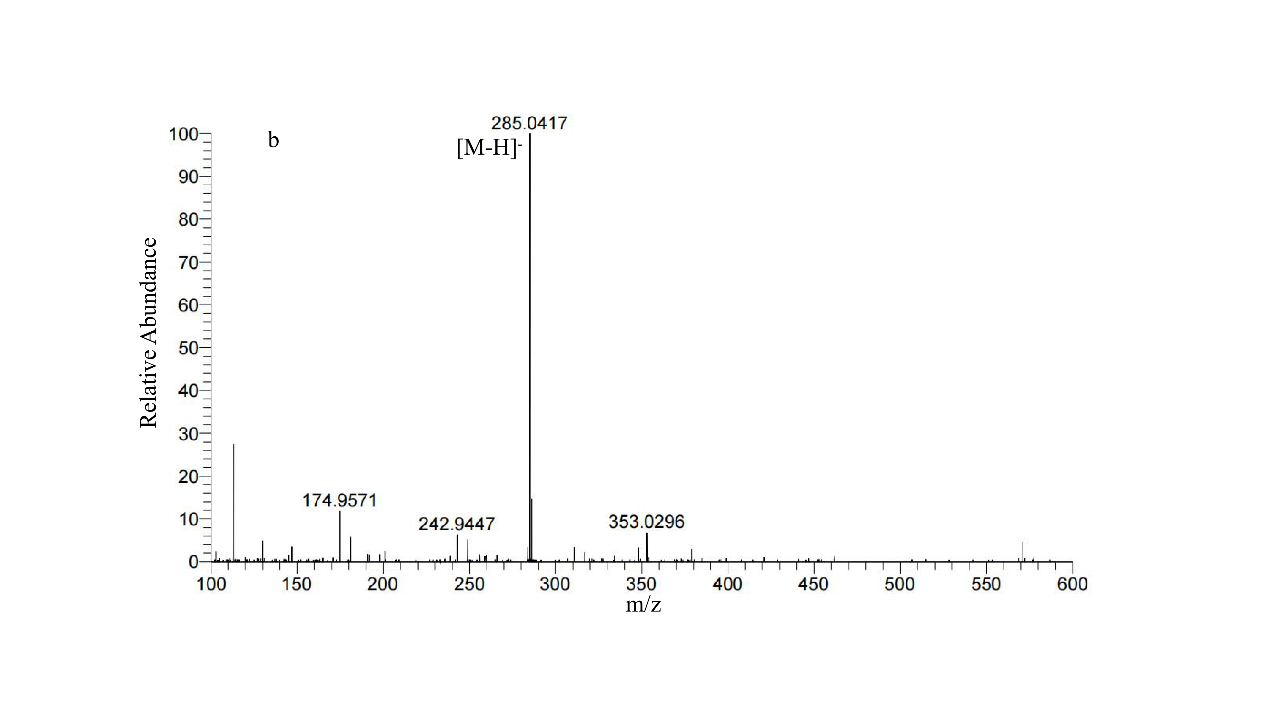
**

**
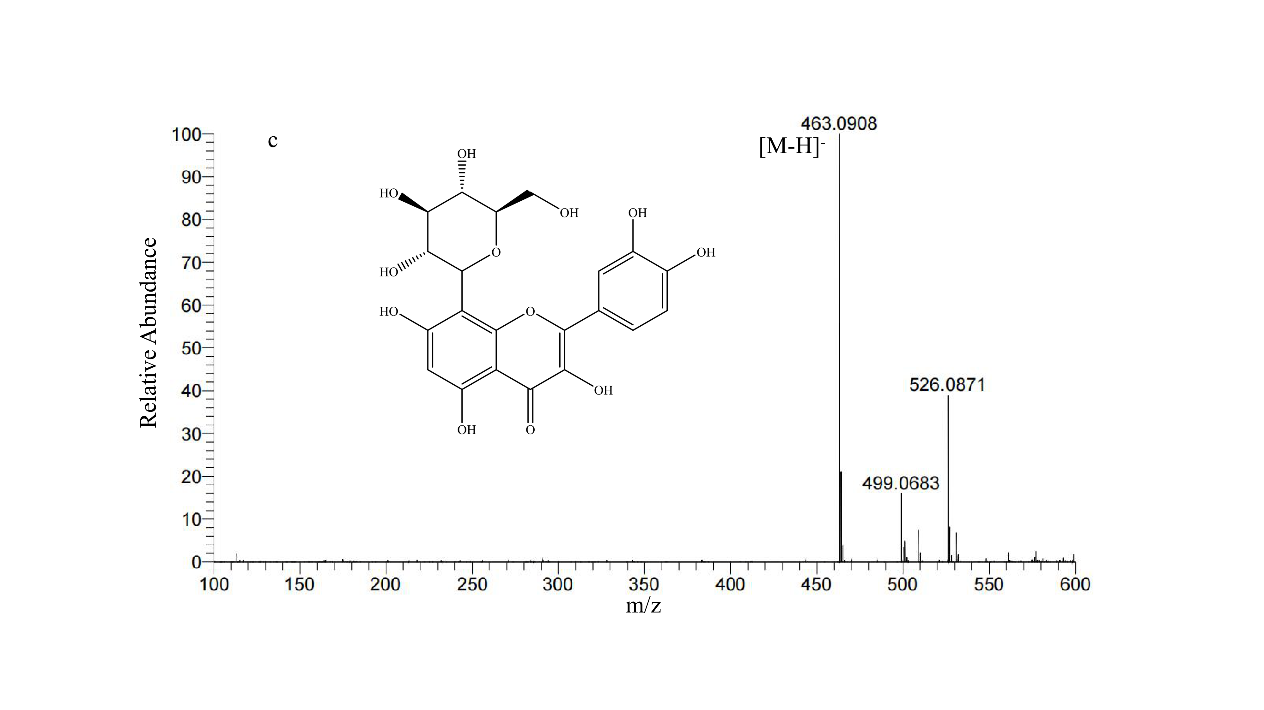
**

**
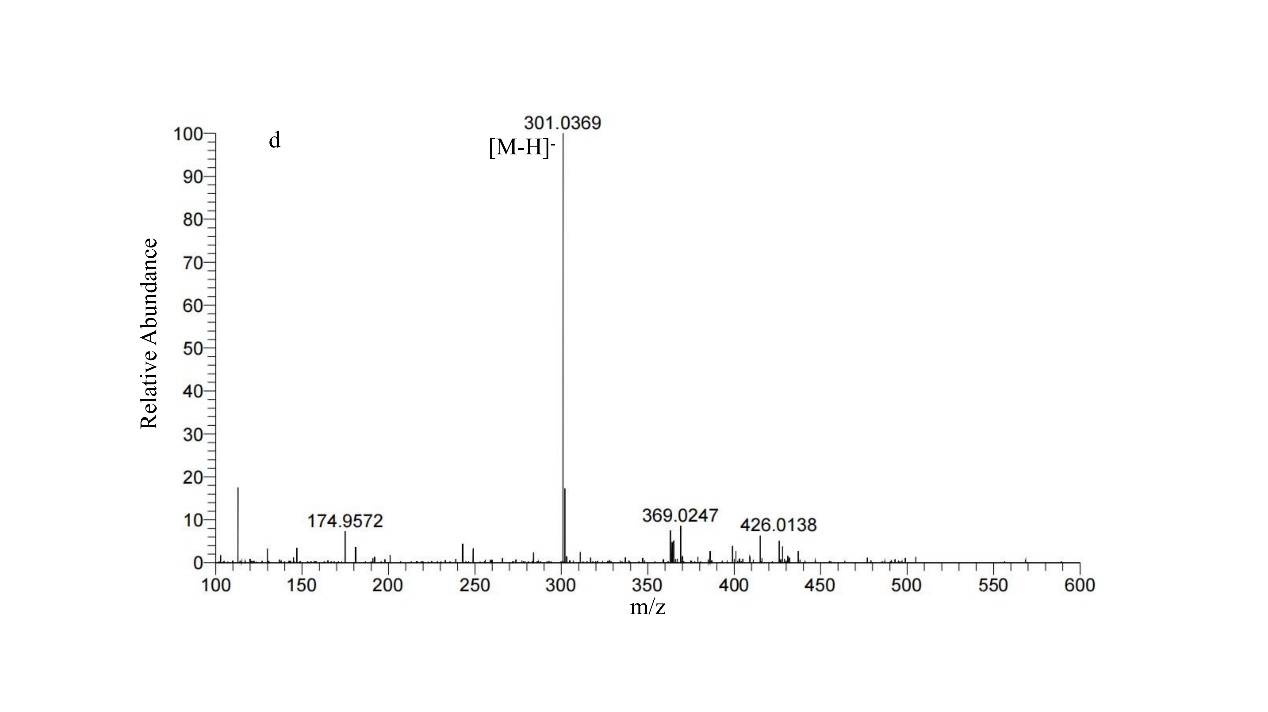
**

**Figure S5.** LC/MS analysis of kaempferol 8-C-glucoside and quercetin 8-C-glucoside production in BL-TcCGT-I. **(**a) kaempferol 8-C-glucoside, (b) kaempferol, (c) quercetin 8-C-glucoside, (d) quercetin.

1. * Corresponding author: Corresponding author: Jianjun Pei. College of Chemical Engineering, Nanjing Forestry University, Nanjing 210037, China. Phone: +86-025-85427962. E-mail: [peijj2000@sina.com.cn](mailto:peijj2000@sina.com.cn). Linguo Zhao. College of Chemical Engineering, Nanjing Forestry University, Nanjing 210037, China. Phone: +86-025-85427962. E-mail: [njfu2302@163.com](mailto:njfu2302@163.com) and [lg.zhao@163.com](mailto:lg.zhao@163.com). [↑](#footnote-ref-1)
